# In-silico screening and analysis of missense SNPs in human CYP3A4/5 affecting drug-enzyme interactions of FDA-approved COVID-19 antiviral drugs

**DOI:** 10.1038/s41598-025-85595-x

**Published:** 2025-01-16

**Authors:** Amro A. Abdelazim, Mohamad Maged, Ahmed I. Abdelmaksoud, Sameh E. Hassanein

**Affiliations:** 1 Department of Pharmaceutical Biotechnology, College of Biotechnology, Misr University of Science and Technology, Giza, Egypt; 2 Applied Biotechnology Program, School of Biotechnology, Nile University, Giza, Egypt; 3 Industrial Biotechnology Department, Genetic Engineering and Biotechnology Research Institute, University of Sadat City, Sadat City, Egypt; 4 Bioinformatics Program, School of Biotechnology, Nile University, Giza, Egypt

**Keywords:** Biochemistry, Biological techniques, Biotechnology, Computational biology and bioinformatics, Genetics, Molecular biology, Structural biology, Biomarkers, Health care

## Abstract

Single nucleotide polymorphisms (SNPs) represent the prevailing form of genetic variations observed in the human population. Such variations could alter the encoded enzymes’ activities. CYP3A4/5 enzymes are involved in metabolizing drugs, notably antivirals against SARS-CoV-2. In this work, we computationally investigated antiviral-enzyme interactions of CYP3A4/5 genetic variants. We also examined the deleterious impact of 751 missense single nucleotide polymorphisms (SNPs) within the CYP3A4/5 genes. An ensemble of bioinformatics tools, [SIFT, PolyPhen-2, cadd, revel, metaLr, mutation assessor, Panther, SNP&GO, PhD-SNP, SNAP, Meta-SNP, FATHMM, I-Mutant, MuPro, INPS, CONSURF, GPS 5.0, MusiteDeep and NetPhos], identified a total of 94 variants (47 SNPs in CYP3A4, 47 SNPs in CYP3A5) to potentially impact the structural integrity as well as the activity of the CYP3A4/5 enzymes. Molecular docking was done to recognize the structural stability and binding properties of the CYP3A4/5 protein isoforms with 3 FDA-approved antiviral drugs. Our findings indicated that the CYP3A4 gene variants; R418T, I335T and R130P and the CYP3A5 gene variants; I335T, L133P and R130Q are considered the most deleterious missense SNPs. These mutants potentially affect drug-enzyme binding and hence may alter therapeutic response. Cataloguing deleterious SNPs is essential for personalized gene-based pharmacotherapy.

**Supplementary Information:**

The online version contains supplementary material available at 10.1038/s41598-025-85595-x.

## Introduction

During the COVID-19 pandemic, several antivirals were tested as treatment candidates in humans^1^. Part of the tested antivirals offered notable recovery rates in some people, while appearing ineffective for others^2^. However, global efforts to investigate the available antivirals and their efficacy in managing COVID-19 disease have been limited by the slow pace of studying the underlying genetic determinants affecting drugs’ metabolism^3^. Remdesivir and nirmatrelvir/ritonavir are antiviral drugs licensed by the FDA to treat SARS-CoV-2^4^. Moreover, they were approved for compassionate use by the European Medicines Agency (EMA) in severe cases. Nonetheless, few studies have reported the underlying pharmacogenetic responses^5^ of these drugs. Human cytochrome P450 (CYP) enzymes have key functions in drug elimination, cellular metabolism, and maintaining homeostasis. Approximately, 80% of oxidative metabolism and over 50% of the total elimination of the commonly used medications is done in the liver. This is attributed to one or more of the cytochrome P450 enzymes, notably, those belonging to the CYP family 1, 2, and 3. Mutations in the genes coding for the cytochrome p450 enzymes have the potential to impact drugs’ responses by altering drugs’ bioavailability^6^. CYP3A4 is responsible for the biotransformation of more than 70% of all prescribed medications^6^. Specifically, CYP3A4/5 enzymes play a pivotal role in metabolizing antiviral drugs targeting SARS-CoV-2 virus^5,7^. Single nucleotide polymorphisms (SNP) in genes encoding CYP3A4/5 enzymes could affect the proteins’ structure and activity. Some polymorphisms are silent^8^, not affecting protein translation, while others are missense SNPs which change the amino acid sequence, thus changing the conformation or the activity of the translated protein^9^. Little is known about the missense SNPs in the genes encoding the CYP3A4/5 drug-metabolizing enzymes. This stems in part from the vast number of genetic variations in the human genome and the incomplete mapping of genetic polymorphisms in some parts of the world. Studying these polymorphisms is useful to develop tailored pharmaceutical interventions. Understanding pharmacogenetics’ factors, such as variations in drug conversion, metabolism, and biotransformation, may help predict therapeutic efficacy and minimizing the likelihood of side effects in patients^10^. Of note, a considerable number of antiviral medications used in the management of COVID-19 lacked comprehensive pharmacogenetics investigations, owing to time constraints^5^ and the overwhelming emergency of the pandemic.

Numerous computational algorithms have been developed to evaluate probable therapeutic outcomes before clinical experiments are conducted to optimize efforts^11^. Currently, in silico technologies can determine the harmfulness of single nucleotide polymorphisms via computational tools^12^. Predicting the effects of mutations is more accurate when multiple algorithms are combined compared to using only one software^13^. In a simulated environment, advanced approaches like molecular docking and molecular dynamics simulations could accurately examine protein features including chemical properties, structure, and molecular interactions^14^. Previous computational studies have elucidated the molecular mechanisms of SARS-CoV-2 disease susceptibility by analyzing high-risk missense variants of some human genes^15,16^. The precision of bioinformatics studies is highly dependent on the quality and quantity of the input data. Unreliable or insufficient data might result in deceptive outcomes, posing a major challenge in the biomedical research field^17^, especially, when results could potentially impact patient’s health and clinical outcome. Thus, it is imperative to validate any used computational algorithms and in-silico tools to improve real-life applicability^18,19^.

Missense single nucleotide polymorphisms in the coding domain of CYP3A4/5 affect the amino acid sequence of the enzymes. It is important to note that not all nonsynonymous missense single nucleotide polymorphisms that alter protein structure and function are harmful. Thus, distinguishing hazardous from benign single nucleotide mutations is critical^9^.

In our efforts to help individualize the anti-COVI-19 medicine, we examined the effects of missense SNPs in genes encoding CYP3A4/5 enzyme isoforms on their structure and activity^5,7^. Moreover, we aimed to distinguish deleterious from benign SNPs, and then perform SNP modelling and molecular docking simulations to predict enzyme-drug interactions using the EMA and FDA-approved antivirals remdesivir and nirmatrelvir/ritonavir ^20,21^.

## Methods

### SNP data retrieval

The ENSEMBL database^22^ provided the CYP3A4/5 SNPs information. CYP3A4 and CYP3A5 SNPs were filtered according to these criteria “*Homo sapiens*, Missense variations, and coding sequence variants”. ClinVar^23^ was used to collect data using the variation type filter (Single nucleotide). Both SNPedia^24^ and PharmVar^25^ were used to aggregate clinically significance data from credible sources. A list of all bioinformatics tools used in this study is shown in Table 1.

**Table 1:** List of all bioinformatics tools used for determination of deleteriousness, stability, and conservation.

Determination category	Tool’s name	Tool’s prediction outcomes	URL	References
Determination of deleteriousness	SIFT	Deleterious/Tolerated	https://sift.bii.a-star.edu.sg	Sim et al.^55^
	PANTHER	Damaging/Benign	http://pantherdb.org/tools/csnpScoreForm.jsp	Tang et al.^56^
	Polyphen-2	Probably damaging/Possibly damaging/Benign	http://genetics.bwh.harvard.edu/pph2/dbsearch.shtml	Adzhubei et al.^57^
	SNPs&GO	Disease/Neutral	https://snps.biofold.org/snps-and-go/pages/method.html	Calabrese et al.^58^
	PHD-SNP	Disease/Neutral	https://snps.biofold.org/phd-snp/phd-snp.html	Capriotti et al.^59^
	CADD	Likely deleterious/Likely benign	https://cadd.gs.washington.edu/snv	Rentzsch et al.^60^
	REVEL	Likely disease causing/Likely benign	https://genome.ucsc.edu/cgi-bin/hgTrackUi?db=hg19&g=revel	Ioannidis et al.^61^
	SNAP	Disease/Neutral	https://www.rostlab.org/services/snap/	Bromberg et al.^62^
	MetaLR	Damaging/Tolerated	https://asia.ensembl.org/info/genome/variation/prediction/protein_function.html#MetaLR	Dong et al.^63^
	MutationAssessor	High/Medium/Low/Neutral	http://mutationassessor.org/r3/	Frousios et al.^64^
	Meta-SNP	Disease/Neutral	https://hub.docker.com/r/biofold/meta-snp	Capriotti et al.^65^
	FATHMM	Damaging/Tolerated	http://fathmm.biocompute.org.uk/	Shihab et al.^66^
Determination of stability upon mutation	MUpro	Decreased stability/ Increased stability	http://mupro.proteomics.ics.uci.edu/	Khan et al.^27^
	I-Mutant v.3.0	Decreased stability/ Increased stability	https://folding.biofold.org/i-mutant/i-mutant2.0.html	Capriotti et al.^26^
	INPS	Decreased stability/ Increased stability	https://inpsmd.biocomp.unibo.it/inpsSuite/default/index	Savojardo et al.^28^
Determination of conservation score	ConSurf	A scale from 0 to 9	http://consurf.tau.ac.il	Chorin et al.^30^
Prediction of post-translation phosphorylation sites	NetPhos 3.1	The amino acid position alongside the predicted kinase enzyme responsible for the phosphorylation event	https://services.healthtech.dtu.dk/services/NetPhos-3.1/	Blom et al.^31^
Identification of protein binding sites	COACH	The binding Residues of the enzyme	https://zhanggroup.org/COACH/	Yang et al.^32^
Modelling of the SNPs	Swiss-Model	PDB files of the modelled SNPs	https://swissmodel.expasy.org/	Waterhouse et al.^33^
Models’ validation	TM-Align	RMSD and TM-Score	https://zhanggroup.org/TM-align/	Zhang et al.^38^
	QMEANDisCo	Sequence identity, QMEAN, C-beta (Cβ), all atoms, solvation, torsion, GMQE and QMEANDisCo Global	https://swissmodel.expasy.org/qmean/	Studer et al.^40^, Benkert et al.^39^
	MolProbity	MolProbity score, Clash score, Ramachandran favoured and Ramachandran Outliers	http://molprobity.biochem.duke.edu/	Chen et al.^41^
Docking	MOE	Scores of docking poses ( Kcal.mol − 1)	https://www.chemcomp.com/Products.htm	Chemical Computing Group et al.^43^
Docking results 2D/3D visualization	BIOVIA Discovery studio	2D and 3D picture representations of all drug-protein docking poses illustrating the ponds and their types	https://www.3ds.com/products/biovia/discovery-studio/	Dassault Systèmes BIOVIA et al.^44^

### In-silico determination of deleteriousness

Twelve in-silico tools were employed to evaluate deleteriousness of the CYP3A4/5 variants: SIFT, PolyPhen-2, cadd, revel, metaLr, mutation assessor, Panther, SNP&GO, PhD-SNP, SNAP, Meta-SNP and FATHMM. For each SNP, a score of 12 was given based on how many tools gave the SNP an outcome that represented deleteriousness (disease causing/ deleterious/ damaging) and non-deleterious results (tolerated, benign, and neutral). For each tool that gave a “deleterious result” the SNP received a “+1 score” and for each “non-deleterious result” the SNP receivess a “0 score”. The MutationAssessor tool provides a unique representation of its results in the form of 4 categories (neutral/low/medium/high) in which only the result “high” was assigned a “+1 score”.

### Stability determination upon mutation

Single amino acid substitutions may alter protein structure. Thus, it is necessary to accurately determine how amino acid changes affect protein stability. A theoretical and experimental investigation of the mutant energy characteristics was conducted. Three in-silico tools [I-Mutant^26^, MuPro^27^, and INPS^28^) were used to determine CYP3A4/5 stability following mutation.

### Evolutionary conservation analysis prediction profile

The evolutionary conservation of an amino acid in a protein indicates a delicate balance between its intrinsic mutability and its necessity to preserve macromolecule structural integrity and functional efficiency^29^. The ConSurf tool was used for this step. Consurf analysis can efficiently and comprehensively examine protein functional regions for high-throughput characterization. A computer model predicts the evolutionary tendencies and phylogenetic preservation of amino acid residues in a protein sequence. The server calculates a conservation score for each residue from 1 to 9. Additionally, the server predicts whether each residue is useful or structural. A numerical scale was used to classify residues in our study. The variable residues are 1–3, whereas typical conserved residues are 4–6. 7–9 residues are well conserved. High conservation and accessibility define functional residues. Alternatively, structural residues can be buried inside the protein structure but are significantly conserved^30^.

### Posttranslational modification (PTM) site prediction

Posttranslational modifications regulate protein biology, therefore finding their sites is crucial. These modifications impact the stability of proteins, influence enzyme activity, and pathogenicity of genetic variations^29^. The tool used for this step was NetPhos 3.1. The service predicts eukaryotic protein serine, threonine, and tyrosine phosphorylation sites using neural network ensembles. Both general and kinase-specific predictions are made^31^.

### Identification of protein binding sites

Identification of protein-ligand binding sites is crucial for protein function annotation and therapeutic development. Biological and medical researchers need accurate prediction of ligand-protein interaction regions. However, a general method for determining optimal binding sites across numerous protein types has not been developed. Combining complementary forecasts is likely the best strategy^32^. COACH tool was used for this step.

**COACH** is a meta-server technique used for protein-ligand binding site prediction. The COACH tool predicts complementary ligand binding sites based on target protein structure using two comparison approaches, TM-SITE and S-SITE. These approaches compare binding-specific substructures and sequence profiles to identify ligand-binding models in the BioLiP protein function database. To anticipate ligand binding sites, the above predictions could be combined with the COFACTOR, FINDSITE, and ConCavity results. Researchers may also insert primary sequence data for the I-TASSER method to create three-dimensional models. The COACH pipeline estimates ligand-binding site locations from these models, allowing researchers additional flexibility^32^.

### Modelling of SNPs

Predicting protein structure is important in biomedicine and pharmaceuticals. Homology modelling, often called comparative modelling, is utilized for identifying protein targets with significant structural similarities. The Swiss-Model web service^33^ was used for modeling the 3D structure of the enzymes with their respective SNPs. PDB templates were obtained from the RCSB Protein Data Bank^34^ (RCSB PDB) Both templates showed biological relevance for use in this study, hence; both included the drug ritonavir as the co-crystalline target. Additionally, both templates showed acceptable resolutions and R values.

For CYP3A4 SNPs: the structure with a PDB ID (3nxu)^35^ was used as a template.

For CYP3A5 SNPs: the structure with a PDB ID (5veu)^36^ was used as a template.

### Model validation

Given the importance of quality evaluation in predictive approaches such as homology modelling, each model must include several quality evaluations. Most homology-modelling programs generate many protein 3D models and score them using various methods. The integration of multiple assessment methods may improve evaluation reliability since each method analyzes the three-dimensional structure from different angles. Some errors may require repeating prior steps; therefore, homology modelling is not usually the end step^37^. TM-Align^38^, QMEAN^39^, QMEANDisCo^40^ and MolProbity^41^ were used for model structure validation before use in docking experiments. The *TM-Align* algorithm is used for aligning and comparing protein structures. A TM-score closer to 1 statistically indicates a “high similarity in fold”. Additionally, the **root mean square deviation (RMSD)** is utilized to measure the similarity between two structures, with a lower RMSD indicating a higher degree of similarity.

The *QMEANDisCo tool* incorporates the validation of “8 distinct elements” in any given model. These elements include **sequence identity**, which represents the similarity ratio between the amino acid sequences of the template structure used to construct the model and the actual final model (with 100% being the optimal value).

The **QMEAN** is a powerful estimator that utilizes various geometric features to provide accurate quality assessments of protein structures. It provides comprehensive assessments for the entire structure as well as specific evaluations for individual residues using a single model.

**C-beta (Cβ)** is a measurement that is affected by the presence of incompatibilities caused by misfit conformations or inappropriate refinement constraints between the sidechain and backbone. These incompatibilities might alter the sensitivity of the C-beta assessment. The **QMEAN Z-scores** category includes additional values, such as the **all-atoms** value, **solvation** value, and the **torsion** value. These five measures are evaluated by comparing them to values obtained from a large collection of empirical structures of similar dimensions, using Z-scores. **The Global Model Quality Estimate (GMQE)** is used to estimate the quality of a model. It evaluates qualities obtained from both the alignment between the target and template sequences, as well as the structural properties of the template. A higher GMQE score indicates better quality. The **QMEANDisCo global** score assesses the overall quality of a model, on a scale from 0 to 1. Higher scores indicate a higher projected quality.

The third validation tool employed was *MolProbity*. It is an online tool that evaluates the quality of protein models by doing a comprehensive and reliable assessment at both the local and global levels of the structure. The tool yields four distinct assessments, the MolProbity score, clash score, Ramachandran favored, and Ramachandran outliers.

### Molecular docking analysis

#### Ligand and protein preparations

First, ligands were obtained from the PubChem database^42^ as follows:

1-Remdesivir PubChem CID: 121,304,016.

2-Nirmatrelvir PubChem CID: 155,903,259.

3-Ritonavir PubChem CID: 392,622.

The ligands were prepared for docking by first being subjected to protonation and subsequently to energy minimization. By doing so, the 3D structures of the ligands become oriented in their most stable conformer possible. Second, water molecules and built-in ligands are removed from the PDB files of the modelled SNPs before docking. Crash reduction is maximized by this approach, optimizing the docking results. The preparation of both ligand and protein files was done using the molecular operating environment software (MOE software). After that, the ligand library was created for docking.

The modelled SNPs PDB file was subjected to similar preparation, where water molecules and extra ligands were removed. The SNPs PDB files were prepared just like the wild-type crystal structures PDB file, before being used in the docking experiment. If the protein files contained multiple subunits of the same crystal structure, any extra subunits were deleted. Similarly, any water molecules, metal ions or extra ligands were removed from the file. Then the proteins were protonated as well.

The docking experiment was carried out using the MOE software by according to its default settings. The docking scores were reported in Kcal/mol unit, and the software BIOVIA discovery studio was used for the 2D/3D visualization of the ligand-protein complexes. The same software was used to calculate RMSD values between any ligand-wild-type docking pose and the same ligand-SNP docking pose then the RMSD values are recorded for further analysis.

### Docking results visualization and analysis

MOE software^43^ was used for the docking process and BIOVIA Discovery Studio 2021^44^ (V21.1.0.20298) was used for visualization of drug-protein 2D/3D interactions and for the root mean square deviation (RMSD) calculations.

### Prediction of protein 3D structure changes upon mutation

Understanding how mutations affected the protein’s three-dimensional structure could explain its activity, phenotype and the cause of the deleterious effect^45^. The tool used in this step was HOPE.

#### HOPE (have our protein explained)

Mutant proteins were analyzed thoroughly by the application. The system gathers data from several sources, including protein 3D coordinate calculations. The server must be given the protein’s amino acid sequence and details on the mutation’s location and kind before performing the analysis^45^.

### Primers designed for future experimental studies

To increase the clinical significance of the study findings, we designed PCR primers that are specific for detecting the identified deleterious SNPs in both the CYP3A4 and CYP3A5 genes via Primer-BLAST Tool (NIH) (Table S17). All bioinformatics tools used in this study are listed in (Table 1).

## Results

In our efforts to examine the CYP3A4/5 missense single nucleotide polymorphisms and their correlation with enzyme-drug interactions, several layers of investigation were done. The objective of these successive layers was to filter out the missense SNPs that may not affect enzyme integrity, activity, regulation or enzyme-antiviral binding. This was further complemented with verification of the missense SNPs that alter antiviral-protein interaction. The first layer involved the preliminarily exclusion of the missense SNPs predicted to cause no disease. This was further followed by structural and conformational stability analysis. Then functional activity and regulation were assessed. Finally, docking simulations were conducted.

### Data retrieval of the missense SNPs

After filtering out data outside our study aims, the ENSEMBL search revealed 415 SNPs for CYP3A4 and 336 for CYP3A5. The ClinVar tool identified 6 SNPs in the CYP3A4 gene namely (P488H, T363M, Y347C, I301T, I193M, N192K) and 11 in the CYP3A5 gene (P466H, P389S, P344Q, T309N, S299A, R260H, P169S, L120S, V93L, Y53H, L46F). All the ClinVar results were already found in the ENSEMBL database. Utilizing SNPedia tool, we identified 14 SNPs in CYP3A4 gene (P467S, M445T, L373F, T363M, L293P, S222P, P218R, F189S, T185S, R162Q, R130Q, I118V, G56D, L15P) and 8 SNPs in CYP3A5 gene (I488T, F446S, T398N, A337T, Q200R, L82R, Y53C, R28C), all of which were previously found in the ENSEMBL database. PharmVar tool discovered 27 SNPs in the CYP3A4 gene (P467S, M445T, I427V, P416L, L373F, A370S, T363M, I335T, H324Q, Y319C, L293P, S222P, P218R, Q200H, F189S, T185S, D174H, V170I, R162Q, R162W, R130Q, I118V, F113I, G56D, L22V, L15P, L3V) and 8 in the CYP3A5 gene (I488T, F446S, T398N, A337T, Q200R, L82R, Y53C, R28C), all of which were previously present in the ENSEMBL.

### In-silico determination of the deleteriousness of the missense SNPs

In our attempts to assess the data output of the 12 programs used to nominate “deleteriousness” of mutations in the CYP3A isoforms, scores were assigned.

#### In CYP3A4

Subjecting all 415 CYP3A4 SNPs to the 12 aforementioned tools came as follows, 78 SNPs had a score of 2 out of 12, 29 SNPs had a score of 3 out of 12, 26 SNPs had a score of 4 out of 12, 18 SNPs had a score of 5 out of 12, 17 SNPs had a score of 6 out of 12, 20 SNPs had a score of 7 out of 12, 34 SNPs had a score of 8 out of 12, 27 SNPs had a score of 0 out of 12), 78 SNPs had a score of 0 out of 12, 78 SNPs had a score of 9 out of 12, 40 SNPs had a score of 10 out of 12, 6 SNPs had a score of 11 out of 12, and only 1 SNP had a score of 12 out of 12.

#### In CYP3A5

**Fig. 1:**
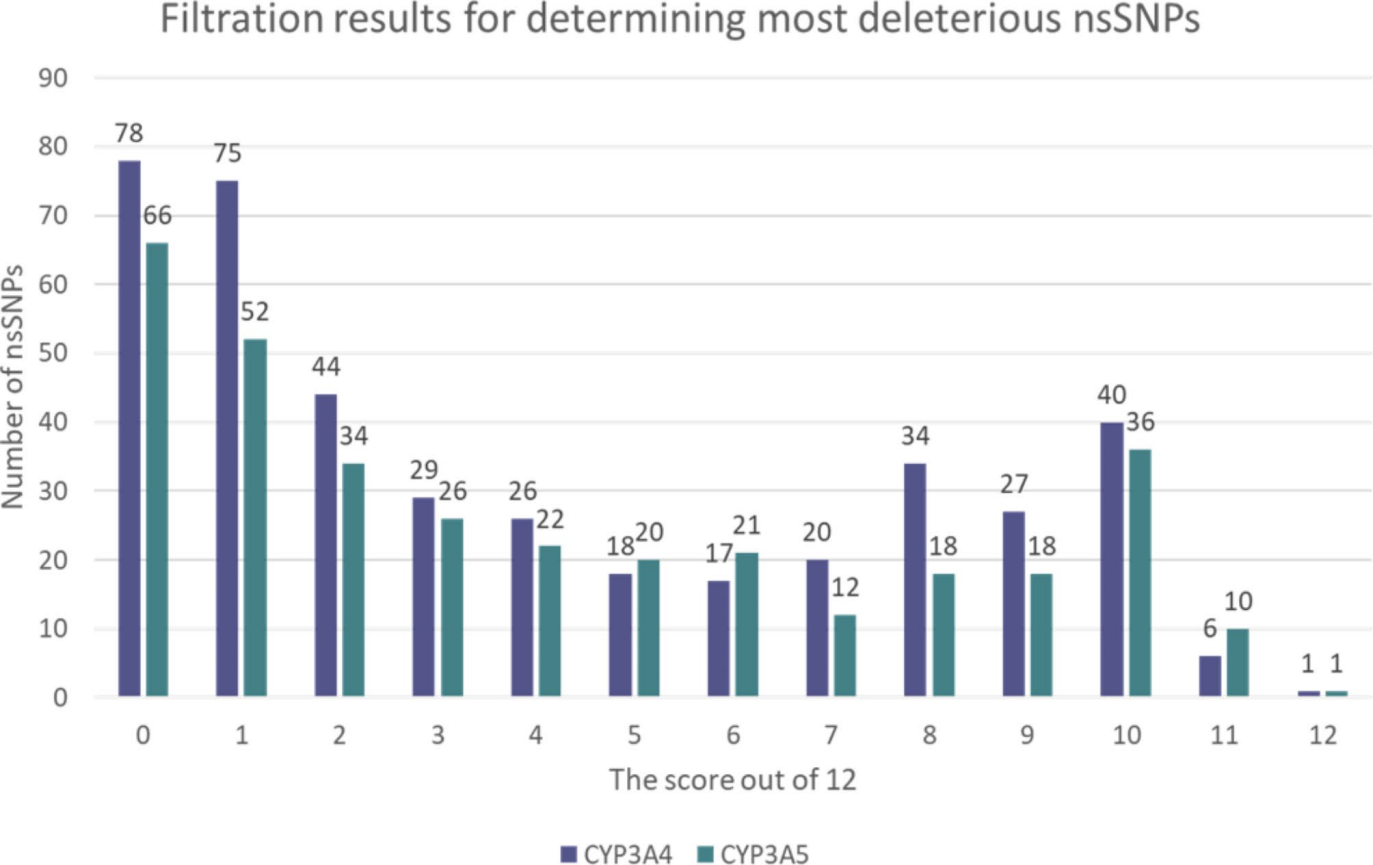
The chart shows the filtration of the CYP3A4/5 most deleterious missense SNPs scoring results.

The outcomes obtained by assigning all 336 SNPs of the CYP3A5 to the 12 specified computational tools were as follows, 66 SNPs had a score of 0 out of 12, 52 SNPs had a score of 1 out of 12, 34 SNPs had a score of 2 out of 12, 26 SNPs had a score of 3 out of 12, 22 SNPs had a score of 4 out of 12, 20 SNPs had a score of 5 out of 12, 21 SNPs had a score of 6 out of 12, 12 SNPs had a score of 7 out of 12, 18 SNPs had a score of 8 out of 12, 18 SNPs had a score of 9 out of 12, 36 SNPs had a score of 10 out of 12, 10 SNPs had a score of 11 out of 12, and finally just 1 SNP had a score of 12 out of 12 as shown in (Fig. 1). To improve reliability of our analysis, It was determined that only SNPs with a score of at least 10 out of 12 would be considered for further investigations, leaving only 47 CYP3A4 SNPs alongside 47 CYP3A5 SNPs (Tables S1 and S2). Additionally, data of “minor allele frequency” of both the CYP3A4 and CYP3A5 variants including variant ID, location of the mutated base pair on the chromosome, global minor allelic frequency are listed in (Tables S3 and S4) respectively.

### Stability determination upon mutation

Three tools were used to assess the structural stability of 47 CYP3A4 and 47 CYP3A5 SNPs following mutation. For the CYP3A4 SNPs, the I-Mutant findings showed that 39 SNPs decreased structural stability following mutation, whereas 9 SNPs enhanced stability. The MU-Pro findings indicated that 42 SNPs lowered structural stability owing to the mutation, whereas 5 SNPs (P416L also known as (CYP3A4*13), P411L, P397L, T310M, K96E) had increased stability. Finally, the INPS data indicated that all SNPs except for P416R and E334K, had decreased stability due to mutation.

As for the CYP3A5 SNPs, the I-Mutant search revealed that 41 SNPs lost stability following mutation, whereas 6 SNPs (P411R, D404Y, Y355C, Y347C, K330E, N104I) gained stability. Nearly every SNP in MU-Pro exhibited a decrease in stability due to the corresponding mutation. However, just one SNP (K330E) potentially increased the stability following the mutation. The INPS study indicated that all the SNPs were less stable following the mutation, except for R418M and S195F, which were putatively more stable. The results of the stability determination of the tabulated mutations for the CYP3A4 and CYP3A5 SNPs are listed in supplementary (Tables S5 and S6) respectively.

### Evolutionary conservation analysis

**Fig. 2:**
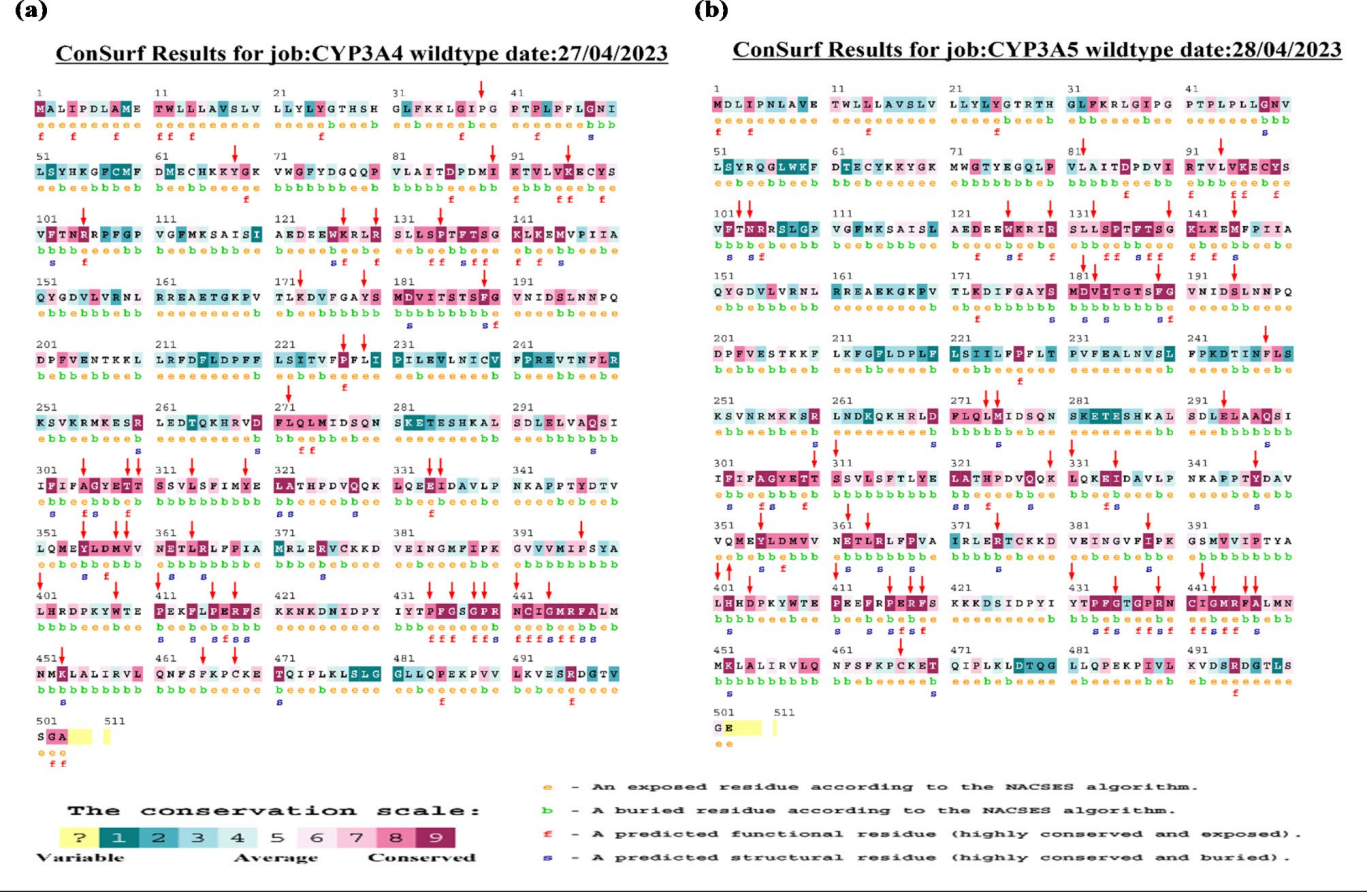
The diagram shows the Consurf results for CYP3A4 (**a**) and CYP3A5 (**b**).

Consurf was used to determine the conservation of each amino acid in CYP3A4/5. The scan could also reveal whether the amino acid is buried or exposed and its importance in the protein’s structure or function. The CYP3A4 Consurf conservation analysis graph is shown in Fig. 2a. The Consurf conservation analysis graph for CYP3A5 is shown in Fig. 2b.

In CYP4A4, nearly all amino acids of the missense SNPs were “conserved-highly conserved” except: C468, F463, L229 which scored “6” and were “averagely conserved” and “P39” which scored “5” and was considered neutral.

In CYP3A5, all amino acids of the missense SNPs under investigation were “conserved-highly conserved” except: Q352, T103, L94, L82 which scored “6” and were “averagely conserved”. Results of the evolutionary conservation analysis for the CYP3A4 and CYP3A5 SNPs can be found in supplementary (Tables S7 and S8) respectively.

### Post translation modification prediction

Posttranslational alterations, mainly phosphorylation^46^, modulate protein stability, activity and are sometimes associated with disease. To identify the mutants that could alter posttranslational modification (PTM) events, NetPhos 3.1 was used. The following predictions have been made for a total of 17 kinases: the ATM serine/threonine kinase (ATM), casein kinase 1 (CKI), casein kinase 2 (CKII), Ca^2+^/calmodulin-dependent protein kinase II (CaMII), the DNA-dependent protein kinase (DNAPK), epidermal growth factor receptor kinase (EGFR), glycogen synthase kinase-3 (GSK3), insulin receptor tyrosine kinase (INSR), protein kinase A (PKA), protein kinase B (PKB), protein kinase C (PKC), protein kinase G (PKG), ribosomal S6 kinase (RSK), proto-oncogene tyrosine-protein kinase Src (SRC), Cyclin-dependent kinase 2 (cdc2), cyclin-dependent kinase 5 (cdk5) and P38 mitogen-activated protein kinases (p38MAPK). If the prediction tool failed to recognize the exact enzyme, the label Unspecified (Unsp) was returned. The NetPhos3.1 program revealed a total of 74 distinct phosphorylation sites for CYP3A4. For the CYP3A5, the tool identified 81 sites influenced by various kinases. The posttranslation modification sites prediction results for CYP3A4 and CYP3A5 are found in supplementary (Tables S9 and S10) respectively.

### COACH “binding site identifier”

An additional layer of analysis was performed to examine the CYP3A4/5 enzyme binding cavity and the effects of amino acid alterations within by using the COACH tool. In addition to identifying enzyme binding sites, the data showed that some mutated residues could have a deleterious effect and were probably residues necessary for the enzyme’s binding site. This finding revealed that the identified SNPs may affect enzyme stability and ligand-enzyme interactions. The heme moiety of cytochrome enzymes binds and interacts with numerous amino acids; therefore any anomalies in this site might affect enzyme activity. Additionally, a matching was done between the amino acids in the “ligand binding site**”** predicted by the COACH server and the amino acids in the reported ritonavir-bound crystal structures.

- **Amino acids of the CYP3A4/ritonavir crystal structure “retrieved from PDB”**: (tyr53, phe57, asp76, arg105, arg106, phe108, met113, ser119, Ile120, leu210, leu211, phe213, phe215, thr224, phe241, Ile300, Ile301, phe304, ala305, thr309, Ile396, ala370, met371, arg372, leu373, glu374, cys442, gly481, leu483).
- **Amino acids matched between the COACH tool and the crystal structure**: (tyr53, phe57, asp76, arg106,ser119, thr224, Ile301, phe304, ala305, thr309, ala370, arg372, glu374)
- **Amino acids of the CYP3A5/ritonavir crystal structure “retrieved from PDB”**: (arg105, arg106, leu108, met114, ser119, leu120, phe210, phe213, gly214, phe215, phe220, leu240, phe241, pro242, Ile300, Ile301, phe304, ala305, thr309, ala370, glu374, cys441, gly480, leu481)
- **Amino acids matched between the COACH server and the crystal structure**: (arg105, ser119, leu120, phe241,Ile301, phe304, ala305, thr309, ala370, glu374, gly480).

Supplementary Tables S11 for CYP3A4 and S12 for CYP3A5 show these amino acids in red to differentiate them. Arg105, Lys127, Arg130, Pro135, Ala305, Thr309, Thr310, Leu364, Pro434, Gly436, Pro397, Asn441 and Gly444 are CYP3A4 amino acids. Leu94, Asn104, Trp126, Arg130, Leu133, Met275, Thr309, Leu364, Arg375, Tyr431, Gly435, Arg439, Ile442, Gly443, Phe446 and Ala447 are in CYP3A5.

### Modelling of SNPs

SWISS-MODEL was used to construct a CYP3A4/5 SNP models for molecular docking and ultimately determine the impact of these SNPs on the binding mode/affinity of the drug. To do so, the predicted models had to be validated and verified for use with confidence. All the 47 CYP3A4 SNP FASTA files alongside all the 47 CYP3A5 SNPs were uploaded to the Swissmodeler with their respective chosen template as described previously. The PDB files for all modelled SNPs were obtained, downloaded and subsequently subjected to further validation steps before application. The model validation results for CYP3A4 and CYP3A5 are shown in supplementary (Tables S13 and S14) respectively.

### SNPs model validation

The validation tools and measurements performed on the CYP3A4 and CYP3A5 mutants’ PDB files indicated that all SNPs models’ authenticity fell within the acceptable values, rendering them suitable for our investigation.

### Molecular docking

The PDB files of the wild-type alongside the SNPs were all prepared for docking as described previously. The software used was molecular operating environment (MOE 2014.0901 i4w9). All steps including drug (remdesivir, nirmatrelvir/ritonavir) preparation, library building, macromolecule preparation and docking were performed using the default program settings. After docking was completed, all docking scores (S scores in Kcal/mol) were retained for examination. For each SNP model, the 3 drugs were docked, each ligand gave 30 poses, and only the pose with the best score for each enzyme-drug complex was taken and recorded, yielding 3 complexes for each SNP to be compared with their wild-type (WT) counterparts. Comparisons of docking scores, enzyme-ligand binding interactions, and docking pose RMSD values were also conducted. The outputs were visualised using Discovery Studio V21.1.0.20298. The exact binding cavity utilized by the MOE software can be presented as follows:

- **CYP3A4/cavity used for docking**: (tyr53 phe57 asp76 arg105 arg106 phe108 met114 ile118 ser119 ile120 trp126 arg130 phe137 ile184 leu210 leu211 phe213 phe215 thr224 phe241 ile300 ile301 phe302 phe304 ala305 gly306 thr309 thr310 val313 leu364 ile369 ala370 met371 arg372 leu373 glu374 arg375 pro434 phe435 gly436 ser437 arg440 asn441 cys442 ile443 gly444 phe447 ala448 met452 gly481 leu482).
- **CYP3A5/cavity used for docking**: (tyr53 arg54 gly56 leu57 trp58 arg105 arg106 leu108 met114 ile118 ser119 leu120 trp126 arg130 phe137 ile184 phe210 leu211 lys212 phe213 gly214 phe215 leu216 phe220 leu240 phe241 pro242 ile300 ile301 phe302 phe304 ala305 gly306 thr309 thr310 val313 leu364 phe367 pro368 val369 ala370 ile371 arg372 leu373 glu374 arg375 thr398 thr432 pro433 phe434 gly435 thr436 arg439 asn440 cys441 ile442 gly443 phe446 ala447 asn450 met451 asp477 thr478 gln479 gly480 leu481 gln483).

Table 2 shows the results of docking analysis of CYP3A4. The ligand-wild-type enzyme docking S scores were obtained as -8.97800922 for remdesivir, -7.20708895 for nirmatrelvir, and finally − 11.5099306 for ritonavir.

**Table 2:** Docking S scores (Kcal/mol) of 47 different CYP3A4 missense SNPs with each of the 3 antiviral drugs.

Variant ID	Macromolecules	Remdesivir	Nirmatrelvir	Ritonavir
Wild Type	CYP3A4	−8.97800922	−7.20708895	−11.5099306
rs1256858657	C468W	−8.87516212	−7.34854221	−11.730319
rs1425544636	C468Y	−9.74769592	−7.10326672	−11.105546
rs71583803	F463C	−9.41393948	−7.07232904	−11.1988535
rs774168721	K453N	−8.97202015	−6.9666934	−11.5102139
rs1162067586	G444A	−9.27137756	−7.26435566	−11.4419041
rs567089575	N441D	−9.11749935	−7.63019466	−11.443902
rs1457998170	P439S	−8.94838333	−6.99418163	−11.7098141
rs1166211708	G438V	−9.13990307	−7.37943792	−11.0079994
rs755248651	G436R	−9.1397686	−7.44101191	−11.0519924
rs1355522141	P434A	−9.03595161	−7.32213879	−11.8670883
rs1368745625	*R418T*	−8.79203033	−7.25248241	*−10.5256042*
rs4986909	P416L(CYP3A4*13)	−9.14723682	−7.18173599	−11.8867626
rs4986909	P416R	−9.30788517	−7.41502571	−11.7398434
rs72552797	P411L	−9.38952637	−7.36195183	−10.6980143
rs1217252102	P411A	−8.95363998	−7.20827532	−10.9971685
rs1044764678	W408R	−8.77373981	−7.16851854	−11.8457041
rs113716682	L401P	−9.62541485	−7.46635723	−11.1873398
rs1481942841	P397L	−9.51131344	−7.12983608	−10.8236246
rs1195408117	L364F	−9.01669884	−7.46199179	−11.2139893
rs71581998	V359E	−8.66892242	−7.36470795	−10.8447914
rs754968125	M358V	−8.93617249	−7.58089447	−11.0206623
rs1201319750	Y355C	−9.30336666	−7.50625706	−11.0144644
rs1462817145	Y355H	−9.12500668	−7.0976181	−12.0400505
rs368296206	I335T(CYP3A4*32)	−9.11065578	*−6.90523386*	−11.1772289
rs867315029	E334K	−8.5060606	−7.47969198	−11.0142565
rs201821708	Y319C(CYP3A4*21)	−9.39177036	−7.35175848	−11.1305771
rs190354371	L314P	−9.50962543	−7.42447996	−11.6400242
rs267601666	L314F	−8.89234734	−7.35286808	−11.0747824
rs751246524	T310M	−9.24810982	−7.63046217	−10.6901493
rs375997724	T309I	−8.95475388	−8.04748154	−11.759696
rs71581996	A305S	−9.39965916	−7.67787838	−11.2086153
rs1368114928	L272P	−9.64168835	−7.92350292	−11.7540007
rs1166537703	L229R	−9.1628294	−7.30100775	−10.6589317
rs1467852216	P227L	−8.80420113	−7.41324663	−11.4226446
rs4987161	F189S(CYP3A4*17)	−9.32750893	−7.71288538	−11.0803633
rs773989431	Y179S	−8.6629324	−7.77411604	−11.276947
rs1396501606	K173E	−8.9990139	−7.69477463	−11.4278498
rs1483230173	P135L	−9.13117599	−7.26505041	−11.9257832
rs72552799	*R130P*	*−8.43525219*	−7.2141099	−10.6372309
rs72552799	R130Q(CYP3A4*8)	−9.16627407	−7.2196517	−11.5641823
rs778013004	R130G	−8.8839407	−7.28858089	−11.1310596
rs1043569086	K127N	−9.16344738	−7.83635426	−11.3305492
rs142296281	R105W	−8.82678986	−7.51242018	−11.2368422
rs3091339	K96E	−9.06612778	−7.4643755	−11.400527
rs1194211831	I90T	−9.57517719	−7.14038897	−10.9687157
rs59418896	Y68C	−9.43817139	−7.49525309	−11.2598972
rs760951972	P39L	−9.24457073	−7.46322584	−11.1436977

SNPs highlighted in italics recorded the lowest docking S score with a ligand.

The wild-type CYP3A4-remdesivir interaction was shown to form one H-bond with Pro434 alongside nine other interactions with residues Ser119, Phe108, Phe435, Leu211, Arg105, Ala370, Cys442 and Ile369 which included interactions such as (Pi-Cation, Pi-Sigma and Pi-alkyl).

The molecular interactions between wild-type CYP3A4 and nirmatrelvir included two H-bonds with Arg105 and Glu374 in addition to other six other interactions with amino acids Arg106, Gly481, Arg372 and Ile369 such as halogen and alkyl interactions.

Finally, wild-type CYP3A4-ritonavir interactions were determined to involve two H-bonds with Gly481 and Thr309 as well as other ten interaction with Ile120, Phe108, Leu482, Ile369, Ala370, Phe215 and Gly481 that included interactions such as Pi-Pi stacked and alkyl interactions). With respect to these previous values, the lowest recorded score for each drug-SNP complex was taken, indicating the least favorable binding/pose energetics.

The R130P mutant had the lowest remdesivir-enzyme docking score which was − 8.43525219 with an RMSD value of 9.5. The macromolecule-ligand interactions were viewed as follows: Two H-bonds with Arg375 and Arg105 neither of which were originally present in the wild-type interactions, nor six other interactions with Glu374, Ala370, Cys442, Leu373, Ile369 and Leu482, missing a total of 3 bonds and losing interactions with residues Ser119, Phe108, Phe435 and Leu211.

I335T (CYP3A4*32) had the lowest nirmatrelvir-enzyme docking S score with a value of -6.90523386 and an RMSD value of 6.4. The drug-enzyme interaction profile had some differences from the wild-type-ligand profile, as it lost its original two H-bonds and gained one new H-bond with Thr309 alongside only three instead of the original six interactions with Phe57, Arg372 and Gly481, in which the halogen interaction was lost.

R418T had the lowest ritonavir-enzyme docking score with a value of -10.5256042 where the SNP-ligand docking pose had an RMSD value of 7.8, and the molecular interactions similarly changed, where two H-bonds were lost and new one formed with Arg105. In addition, there was an introduction of 2 different Pi-anion and Pi-cation interactions that originate from the wild-type complex.

Table 3 presents the CYP3A5 docking results of the wild-type CYP3A5 drug interactions. Docking S scores were calculated as -9.65451717 for remdesivir, -7.38358116 for nirmatrelvir, and − 12.08815 for ritonavir.

**Table 3:** Docking S scores (Kcal/mol) of 47 different CYP3A5 missense SNPs with each of the 3 antiviral drugs.

Variant ID	MUTATIONS	Remdesivir	Nirmatrelvir	Ritonavir
Wild Type	CYP3A5	−9.65451717	−7.38358116	−12.08815
rs777196351	C467R	−9.26423359	−7.93054295	−11.1935425
rs147472467	A447V	−9.08979893	−7.86884785	−11.2487106
rs41279854	F446S(CYP3A5*10)	−8.86362171	−7.54759216	−11.3479376
rs377454308	G443S	−8.76866627	−7.22950363	−10.8261738
rs1267703650	I442T	−9.06812668	−7.72414684	−11.2773447
rs13220949	R439K	−9.65742111	−7.82623291	−11.3792534
rs1035394246	G435E	−8.59579849	−7.29739571	−10.9240675
rs780390510	G435R	−8.83822632	−6.90550661	−11.3247881
rs746993664	Y431N	−8.60551167	−8.33456516	−11.1523771
rs1474237861	F419L	−8.95180988	−7.90459824	−10.9127369
rs370299887	R418M	−9.52617264	−7.54435492	−11.3868389
rs140521496	P416S	−8.66615295	−7.52019644	−10.7156954
rs1299406057	P411R	−9.03088856	−7.19507933	−11.1320887
rs756677833	P411S	−8.79592228	−7.98913145	−11.1473122
rs1562985172	D404Y	−9.09581757	−7.86810684	−10.7338724
rs1252465240	L401P	−9.27143955	−8.06336498	−10.7790775
rs750222754	I388T	−8.73398209	−8.23378086	−10.5891399
rs756271054	R375G	−8.5109787	−7.21004105	−11.3860884
rs779306884	L364H	−8.92117691	−8.06557083	−10.9377079
rs1245832664	E362G	−9.08549881	−7.832757	−10.7698622
rs149888520	Y355C	−9.39531708	−7.32236052	−11.1453447
rs1219950418	Q352P	−8.76727009	−8.6566143	−10.9439602
rs1363320186	Y347C	−8.79192352	−7.93011045	−10.7511187
rs990544214	I335T	−9.25522327	−6.9040904	−11.6457415
rs775816439	L331P	−8.75096607	−6.6627593	−10.8005762
rs1489670280	K330E	−9.0237484	−7.3896451	−11.245019
rs991122268	S311R	−9.02309418	−8.05341053	−10.796299
rs766695006	T309I	−8.86887169	−8.20948505	−10.9470053
rs766695006	T309N	−9.04520416	−7.88267946	−11.7932673
rs1562991842	E294K	−8.91943645	−7.27539349	−11.215642
rs753396001	M275R	−8.85810661	−8.01130962	−11.1118031
rs756839053	L274P	−8.99632549	−7.67974806	−11.216917
rs968643967	F248C	−8.49967003	−7.25709724	−10.9688463
rs979438885	S195F	−9.06109238	−7.40560818	−11.200037
rs1215839981	S195P	−8.35877037	−8.01739788	−11.1082697
rs781765557	F189L	−9.18432999	−8.20416546	−10.7360172
rs777252753	V183G	−8.86363411	−7.89450598	−10.8802357
rs1584446515	D182N	−8.94526958	−7.59848833	−11.3262444
rs764576184	M145T	−8.76924324	−6.90516806	−11.0257168
rs764608096	G140R	−8.65877342	−8.09361935	−11.0615625
rs1383200930	L133P	−8.26592636	−7.89073086	−10.7406998
rs758037875	R130Q	−8.92642307	−7.50071383	−10.5746098
rs1294331881	W126R	−8.29303169	−7.0558629	−10.9266157
rs1186517408	N104I	−9.04933929	−7.63205004	−10.8010502
rs761882111	T103K	−8.88313389	−7.4201951	−11.0002556
rs752003788	L94Q	−8.89486027	−7.29991913	−10.92062
rs56244447	L82R(CYP3A5*3D)	−9.14949512	−7.49991035	−11.0734682

The Wild-type CYP3A5 remdesivir interactions were identified as follow: four H-bonds with Arg105 (3 bonds) and Glu374, with additional seven other interactions with Ile371, Leu481, Ala447, Val369, Pro433 and Cys441 (Pi-alkyl and alkyl interactions). The wild-type CYP3A5 nirmatrelvir molecular interactions included: two H-bonds with Arg105 and Cys441in addition to seven other interactions with amino acids Ala305, Phe304, Ala370, Phe302, Arg105 including Pi-alkyl and alkyl interactions without halogen interactions. The wild-type CYP3A5 ritonavir interactions were shown to have only one H-bond with Cys441 as well as fifteen other interactions: Cys441, Ala305, Phe302, Ala447, Phe446, Arg105, Ile118, Ala370, Phe210, Leu108, Phe220, Phe213 and Leu240 that included Pi-Pi T-shaped, alkyl, Pi-sulfer and Pi-cation interactions.

To evaluate the effect of the SNPs on ligand-enzyme interactions, the minimum recorded S score was determined for each of the drug-SNP complexes. The docking score for the remdesivir-enzyme complex was the lowest for the L133P variant with an S score of -8.26592636 and an RMSD value of 8.057. A complete loss of the conventional H-bonds in the molecular interactions profile was observed alongside the introduction of previously non-existent Pi-anion, Pi-cation interactions. There are ten hydrophobic interactions with Arg439, Arg105, Ser119, Phe220, Leu240, Phe213, Glu374, Ile371 and Ala370. Moreover, the original wild-type interactions with Leu481, Ala447, Val369, Pro433 and Cys44 were lost.

The variant I335T exhibited the most unfavourable docking S score for the nirmatrelvir-enzyme complex, yielding an S score of -6.9040904. Additionally, the variant had an RMSD value of 3.9. The drug formed with the enzyme three H-bonds at Cys441, Ser119 and Glu3374. Losing one original wild-type H-bond and gaining two non-original bonds. Moreover, nirmatrelvir formed eight other interactions with Leu481, Leu373, Arg105, Arg372, Phe302, Ala305 that included two non-original halogen interactions. A total of one interaction was observed when compared to that of the wild-type, and interactions with both Phe304 and Ala370 residues were lost.

**Fig. 3:**
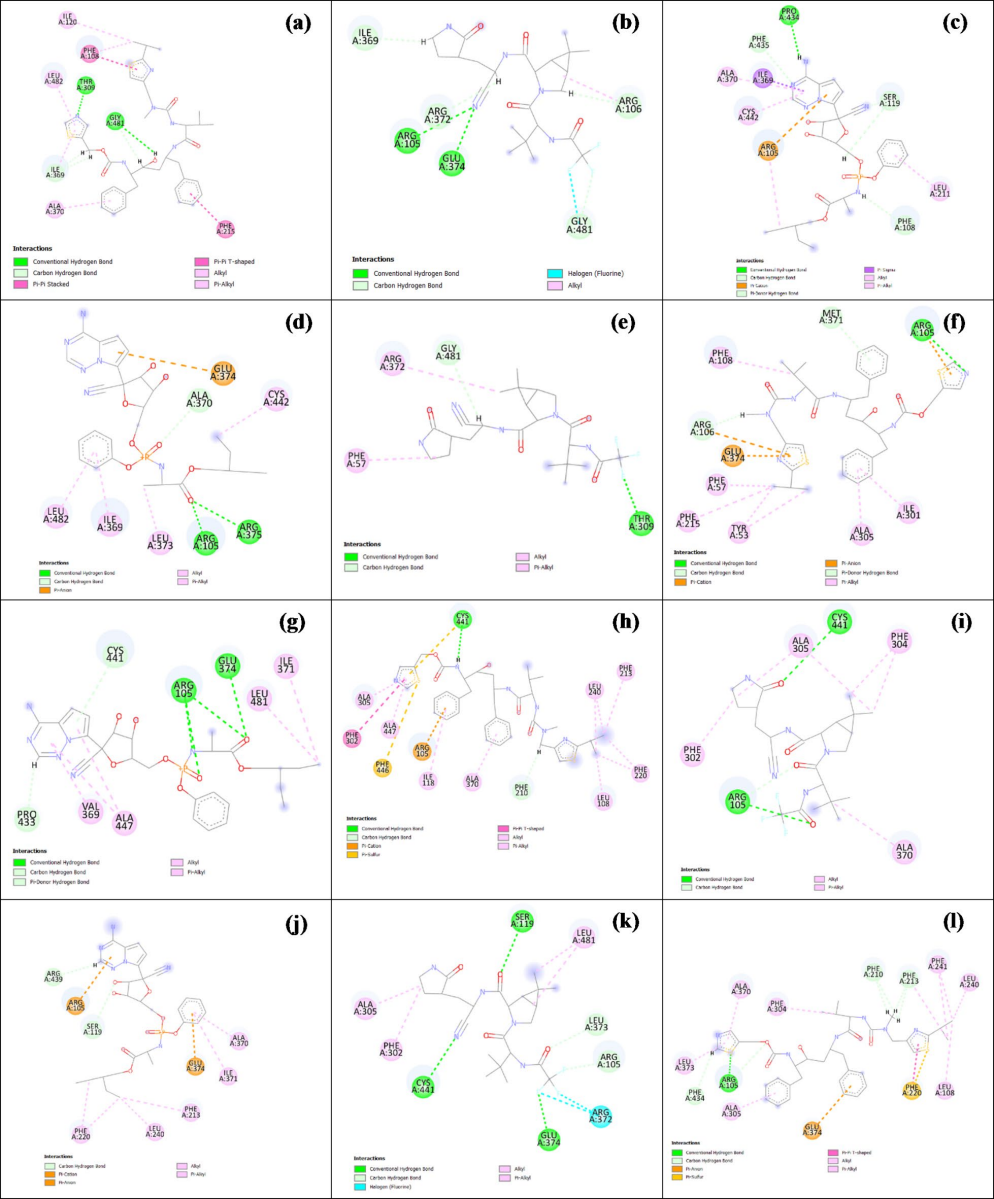
Drug-protein docking poses of wild-type CYP3A4/5 and their mutants showing the lowest docking scores. (**a**) remdesivir docking pose with CYP3A4-Wild-type (**b**) nirmatrelvir docking pose with CYP3A4-Wild-type (**c**) ritonavir docking pose with CYP3A4-Wild-type, (**d**) remdesivir docking pose with CYP3A4- R130P (**e**) nirmatrelvir docking pose with CYP3A4- I335T[*32] (**f**) ritonavir docking pose with CYP3A4- R418T, (**g**) remdesivir docking pose with CYP3A5-Wild-type (**h**) nirmatrelvir docking pose with CYP3A5-Wild-type (**i**) ritonavir docking pose with CYP3A5-Wild-type, (**j**) remdesivir docking pose with CYP3A5-L133P (**k**) nirmatrelvir docking pose with CYP3A5-I335T (**l**) ritonavir docking pose with CYP3A5-R130Q.

The docking S score of the R130Q mutation for the ritonavir-enzyme complex was found to be -10.5746098, indicating the lowest docking score. Additionally, the RMSD value was measured to be 6.4. The interactions were observed to have only one H-bond with Arg105 unlike in the wild-type which was formed with Cys441. There were eighteen other hydrophobic interactions, which meant that 3 interactions were more common than the total in the wild-type. These were formed with Ala305, Arg105, Phe434, Leu373, Ala370, Phe304, Phe210, Phe213, Phe241, Leu240, Leu108, Phe220, Glu374. Interactions with Cys441, Phe302, Ala447, Phe446, Ile118 and Leu108 were lost. Interactions of wild-type CYP3A4/5 drugs and the mutant CYP3A4/5 drugs are shown in Fig. 3. The RMSD values for CYP3A4 and CYP3A5 docking results can be found in supplementary Tables S15 and S16 respectively.

### Structural changes

The software HOPE was used to determine the structural changes associated with the mutations in the most deleterious enzyme SNPs, the ones that had the lowest docking S scores. The tool was employed to evaluate the implications of any amino acid mutation on the overall structure and function of the protein.

#### In CYP3A4 SNPs

With respect to the R418T SNP, substituting arginine with threonine at position 418 was observed to cause the loss of 4 H-bonds present in the wild-type with glutamic acid at position 362, threonine at position 409, glutamic acid at position 410 and lysine at position 413. Additionally, this substitution caused the loss of 3 other salt bridges with glutamic acid at position 362, glutamic acid at position 410 and glutamic acid at position 412. For the SNP I335T(CYP3A4*32), substituting isoleucine with threonine at position 335 appears to cause loss of one H-bond present in the wild-type with glutamine at position 332 as well as another H-bond with leucine at position 339. Additionally, two hydrophobic interactions with leucine at position 460 were lost. In the CYP3A4 variant, R130P, the arginine residue was replaced by proline at position 130. The mutation was predicted to cause loss of two H-bonds with asparagine at position 441 which were formed in the wild-type.

#### In CYP3A5 SNPs

**Fig. 4:**
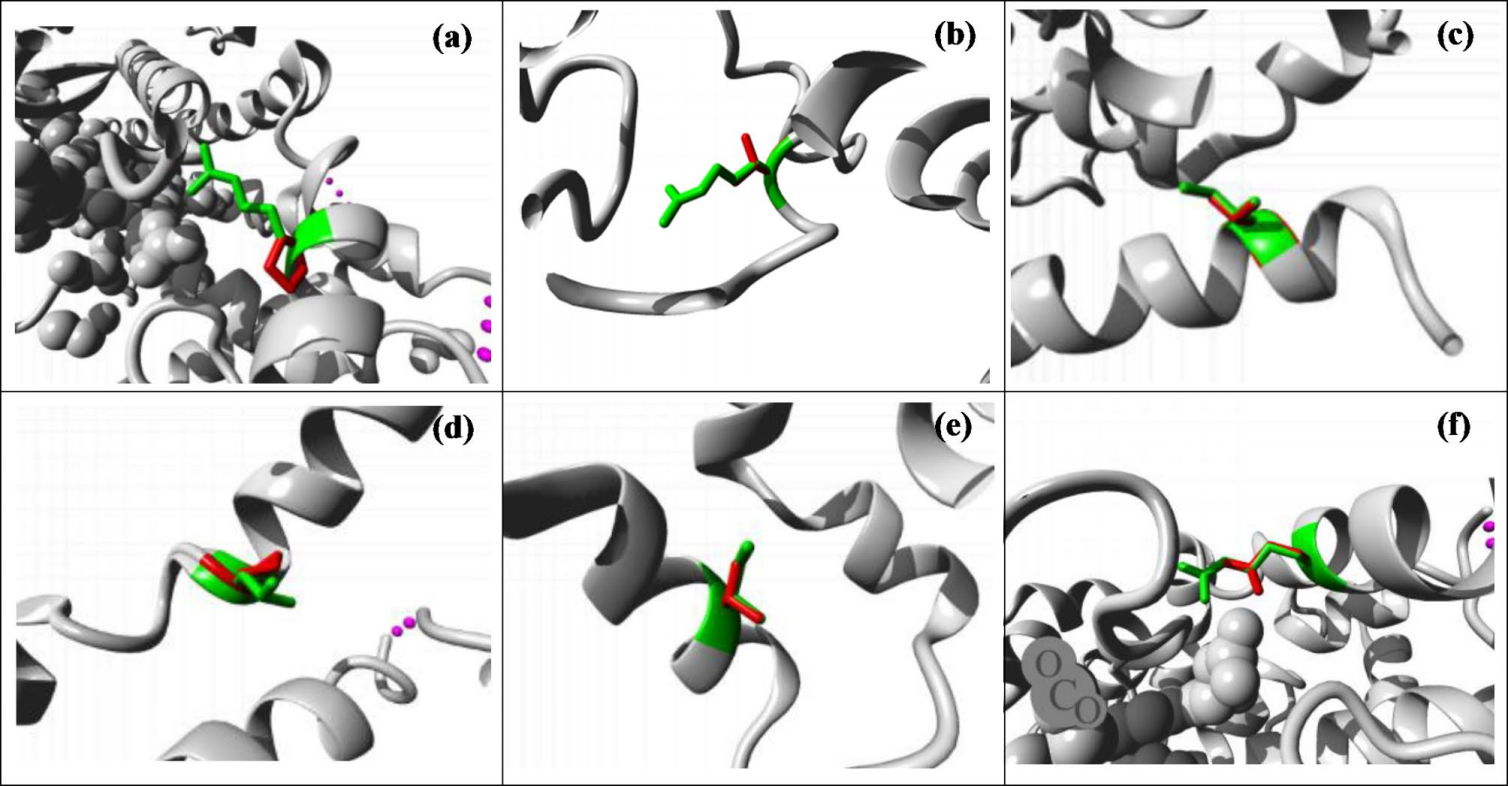
Structural changes associated with mutations of the CYP3A4/5 variants showing the lowest S score with the 3 tested antiviral drugs. (Wild-type residues are colored in green and the mutated residues in red). (**a**) CYP3A4-R130P-Arginine into Proline at position 130. (**b**) CYP3A4-R418T-Arginine into Threonine at position 418. (**c**) CYP3A4-I335T[CYP3A4*32]-Isoleucine into Threonine at position 335. (**d**) CYP3A5-L133P-Leucine into Proline at position 133. (**e**) CYP3A5-I335T-Isoleucine into Threonine at position 335. (**f**) CYP3A5-R130Q-Arginine into Glutamine at position 130.

The I335T mutant, in which the isoleucine residue changed to threonine at position 335 was shown to cause the loss of 1 H-bond with methionine at position 353 as well as another H-bond with valine at position 338. Additionally, the mutation caused the disruption of 7 hydrophobic interactions with methionine at position 353, leucine at position 331, leucine at position 459 and isoleucine at position 456. With respect to the CYP3A5 variant, L133P, It was observed that changing leucine into proline at position 133 caused the loss of a H-bond with isoleucine at position 129 and 6 hydrophobic interactions with phenylalanine at position 302, phenylalanine at position 271 and methionine at position 275. This mutation also caused an empty space in the interior of the protein. Finally, the CYP3A5 variant R130Q, arginine changed to glutamine at position 130. The wild-type residue formed 3 H-bonds with asparagine at position 440 which were lost after the mutation. Figure 4 illustrates CYP3A4 and CYP3A5 structural changes caused by these mutations.

## Discussion

Emerging antiviral medications licensed for use against SARS-CoV-2 are becoming more commonly prescribed in healthcare facilities. Unfortunately, such antiviral medications exhibit marked variations in both their efficacy and safety profiles. Therefore, it is suggested that COVID-19 medications, like many other pharmaceuticals, follow the longstanding principle that “a single prescription does not universally apply to all individuals”. This variation in therapeutic response could be attributed to many factors such as drug bioavailability, concomitant administration of other drugs, underlying genetics, suffering from chronic diseases, age and how the drug is metabolized. Limited data are available concerning remdesivir metabolism as it was first licensed by the FDA for use in May 2020. Despite the subsequent FDA approval of several anti-SARS-CoV-2 medications, few pharmacogenomics studies investigated the impact of cytochrome genes’ nonsynonymous missense single nucleotide polymorphisms on antiviral drugs interactions.

In our work, a multilayered pharmacogenomic framework was used to examine the interactions between 3 first-line antiviral drugs (remdesivir and nirmatrelvir/ritonavir) and the enzymes involved in their metabolism. This approach was done to explain the variation in therapeutic response and safety profiles of such antiviral drugs in COVID-19 patients. Identifying the pharmacogenomics determinants is essential to customize patients’ prescriptions and to optimize therapeutic outcomes. To this end, we undertook a comprehensive multistep in-silico approach to investigate the deleteriousness of missense SNPs of two crucial enzymes involved in the metabolism of 3 key antivirals used with COVID-19 patients. These two enzymes namely, CYP3A4 and CYP3A5, are CYP450 hepatic microsomal enzymes.

A set of 12 benchmarked computational instruments were used to evaluate the deleteriousness of all SNPs under investigation. We adopted stringent criteria; a mutation qualifies as deleterious if picked by at least 10 out of the 12 tools employed. A total of 47 SNPs in the CYP3A4 gene, out of a pool of 415 SNPs, were identified as having deleterious effects. Similarly, 47 out of the 336 SNPs in the CYP3A5 gene were also shown to be deleterious. This indicates that nearly 88% of the purported nonsynonymous missense mutations are not harmful. Thus, we created a library containing potentially deleterious mutants for subsequent investigations. Of note, previous studies predicted the deleterious missense SNPs in CD-209 and HPPD genes using an alternative approach. Filtration of deleterious SNPs was based on scores given by SNPs evaluating servers and p-value^47,48^.

In our efforts to unravel the putative underlying mechanisms of action of these SNPs, a thorough bioinformatics analysis was conducted. Determining the stability after mutation was the subsequent stage in verifying the deleteriousness of the selected SNPs and understanding their effects. Three methods were employed to assess the stability of the 47 CYP3A4 and the 47 CYP3A5 SNPs following mutation. The I-Mutant data showed that 39 CYP3A4 SNPs decreased in stability following mutation, whereas 9 had enhanced stabilities (C468Y, G444A, P416L(CYP3A4*13), P416R, P411L, Y355C, Y319C, T310M, P39L). The MU-Pro findings indicated that 42 SNPs exhibited decrease in stability owing to the mutation, whereas 5 SNPs (P416L (CYP3A4*13), P411L, P397L, T310M and K96E) exhibited increased stability. Finally, the INPS data indicated that all the SNPs decreased the stability of the mutants, except for P416R and E334K, which increased in score. Taken together, these findings indicated that an overall of 39 out of 47 point mutations were deemed deleterious owing to a marked reduction in enzyme conformation and stability upon mutation.

The I-Mutant output analysis revealed that 41 CYP3A5 SNPs lost stability following mutation, whereas 6 SNPs (P411R, D404Y, Y355C, Y347C, K330E and N104I) gained stability. Nearly every SNP in MU-Pro output exhibited a decrease in stability due to the mutation. However, following the mutation, just one SNP (K330E) increased in stability. The INPS study indicated that all SNPs were less stable following the mutation, except for R418M and S195F, which were predicted to be more stable. Thus, collectively, a total of 41 out of 47 point mutations were annotated as deleterious because of the apparent reduction in protein stability. Our data suggested a different mechanism of action of the deleterious effect of the mutation other than molecular stability in the mutants speculated to have gained stability.

Evolutionary conservation analysis using ConSurf servers revealed that nearly all amino acids of the missense SNPs under study were “conserved-highly conserved” with the exception of CYP4A4 “P39L” in which the proline39 was assigned a score of “5”, which indicates “neutral”. It is sensible that nearly all residues under investigation are highly conserved because mutations that occur in a conserved region of a protein could be logically expected to cause detrimental outcomes for the protein conformation and/or function^46^ .

An additional layer of analysis was performed to predict the impact of the missense SNPs on posttranslational alterations and reveal the cause of deleteriousness of such SNPs. To this end, a consortium of 3 programs, namely, GPS 5.0, MusiteDeep and NetPhos, were used. For CYP3A4, variants Y355, Y319, T310, T309, Y179, and Y68 were predicted to cause disturbed posttranslational modification (PTM). Whereas for CYP3A5 SNPs isoforms: Y431, Y355, Y347, S311, T309, S195, and T103 were predicted to have disturbed PTM. This could be accounted for by the observation that all of the designated amino acids are projected to undergo phosphorylation, whereby mutations may cause PTM dysregulation and impact protein stability and function. This aligns with previous research reporting disease-causing mutations in residues involved in cytochrome C phosphorylation^49^.

To deeply analyze the root cause of the deleterious effect of the selected SNPs, molecular docking was done for each mutant and the 3 studied antiviral drugs. Each of the wild-type protein-drug complex could have several predicted poses and the pose yielding the highest docking S score for the given drug was selected as a reference. This was done to compare the molecular interactions and bonding between the wild-type and mutant proteins in a natural simulation of protein-drug associations. The mutants showing the lowest docking S scores were further selected to correlate the structural changes associated with the mutations in addition to, dissecting the intermolecular interactions and bonding as potential causes of deleteriousness.

For the CYP3A4 SNP, R418T, the apparent loss of interactions could arise from the observation that the mutant residue was smaller in size than the wild-type residue, in addition, the wild-type residue was positively charged, and the mutant residue was neutral. Furthermore, we noticed that the mutant residue was more hydrophobic than the wild-type one. All these factors could contribute to placing the amino acid residue away from the correct position to make the same H-bonding necessary for protein integrity as in the original wild-type moiety. This misplacement could also disrupt the ionic interactions of the original wild-type residue, affecting drug (ritonavir) binding and metabolism. Of note, ritonavir is a cytochrome inhibitor that is coadministered with nirmatrelvir to give it enough time to exert its activity^21^. If the SNP could interfere with the inhibitor activity (ritonavir), the nirmatrelvir may be considerably less effective or a subtherapeutic response could be anticipated.

In the I335T (CYP3A4*32) isoform, isoleucine was replaced by threonine at position 335. The mutant residue was smaller in size than the wild-type and also less hydrophobic than the wild-type. This could cause an empty space in the interior of the protein as well as loss of hydrophobic interactions in the core of the protein. In fact, our results with respect to this SNP aligned with previous findings by Hu et al.^50^ and Fang et al.^51^ who stated that CYP3A4 variant I335T was a deleterious mutation that had the potential to damage the protein’s function and hence could affect the metabolism of nirmatrelvir. Notably, nirmatrelvir is a substrate of both CYP3A4/5, and heavily metabolized by them, so it should be coadministered with a powerful CYP inhibitor like ritonavir.

In the CYP3A4 SNP, R130P, arginine changed to proline at position 130. The mutant amino acid was smaller than the wild-type residue, which could cause a distorted orientation of the molecule. Moreover, the wild-type residue was positively charged whereas the mutant residue was neutral and more hydrophobic. Of note, the wild-type residue had interactions with the heme moiety of the cytochrome enzyme. Such interaction is paramount for enzyme function. The difference in charge could affect H-bond formation and ligand association. The wild-type residue was in an α-helix. Proline disrupts the α-helix when not located at one of the first 3 positions of that helix^52^. When the mutation occurs, the helix could be deformed, and this could have severe effects on the structure of the protein. The wild-type residue was highly conserved, and based on conservation scores this mutation is most likely damaging to the protein. Furthermore, the mutated residue was in the binding domain. The difference in the chemical and physical properties between the wild-type and the mutant residues could interfere with the binding affinity to the ligand drug remdesivir. Of note, the antiviral remdesivir was originally used to manage Ebola virus infection and during the COVID-19 pandemic was repurposed to treat the SARS-CoV-2 virus. Remdesivir is a prodrug that becomes activated intracellularly into the triphosphate nucleotide analogue to inhibit viral RNA-dependent RNA polymerase. Cytochrome P450 (mainly CYP3A4) bioconversion of remdesivir has a marked influence on its pharmacokinetic behaviour. Reciprocally, remdesivir is an inhibitor of CYP3A4^53^. Thus, the R130P mutant is flagged for further clinical monitoring in COVID-19 patients.

Among the CYP3A5 SNPs, the variant L133P had a leucine change to proline at position 133. An empty space in the core of the protein was formed because of differences in size and hydrophobicity between the wild-type residue and the mutant one, which is possibly the cause of the loss of hydrophobic interactions required for proper folding of the protein. It could also be observed that the wild-type residue was located in an α-helix^52^. Proline disrupts an α-helix when not located at a particular position on that helix. When the mutation occurs, the helix may be potentially disturbed, and this could have marked adverse effects on the structure of the protein. The mutated residue was located within the catalytic domain and hence it stands to reason that the mutant enzyme may not metabolize remdesivir properly. Thus, this mutation could be of clinical consequence, necessitating watchful monitoring of the administered remdesivir dose and response.

Among the CYP3A5 SNPs, I335T, isoleucine changed to threonine at position 335. The mutant residue was smaller in size and less hydrophobic than the wild-type which could cause a void in the core of the protein as well as loss of hydrophobic interactions required for stabilization. Thus, this SNP could render the nirmatrelvir metabolism, and consequently the therapeutic response, unpredictable.

Finally, CYP3A5 variant R130Q, arginine substituted glutamine at position 130. It is noted that the mutant residue was smaller than the wild-type residue and was located on the surface of the wild-type protein. This could possibly cause loss of external interactions. The wild-type residue was positively charged, whereas the mutant residue was neutral. This loss of charge can cause disrupted molecular interactions. Considering the observed structural damage associated with the mutation, we expected to obtain deviated docking results of the variant SNPs when compared to the wild-type score. The wild-type residue interacts with the heme moiety which is a crucial part of the cytochrome P450 enzyme^54^. The difference in physical and chemical properties between the wild-type and the mutant could interfere with enzyme activity or ligand binding and hence drug (ritonavir) metabolism, rendering the response of the combined treatment (ritonavir/nirmatrelvir) unanticipated.

Our study is the first comprehensive in-silico analysis of CYP3A4/5 missense SNPs and their effects on the enzyme-drug interactions of FDA/EMA-licensed COVID-19 antiviral drugs. Our findings provide valuable insights into the molecular mechanisms of drug metabolism and could help guide the clinical decisions regarding drug choice, dosing regimen and individualized therapy. To this end, we have created a library of oligonucleotide primers to detect the most deleterious gene variants in further PCR tests or diagnostic toolkits. Our work demonstrates the value of in-silico methods to pharmacogenetics research, especially in the context of a pandemic where rapid and reliable information is needed.

This pharmacogenetics study identified deleterious point mutations in the genes encoding CYP3A4/5 enzymes involved in the metabolism of FDA/EMA-approved COVID-19 antiviral drugs, remdesivir, nirmatrelvir/ritonavir. A multistep in-silico pipeline was employed to study the underlying mechanisms of the deleterious effects of these mutations and their impact on drug biotransformation. Structural modelling and conformational analysis combined with docking revealed that putative folding, conformation, drug-binding and catalytic activities of the CYP3A4/5 mutants were notably different from those of the wild-type enzymes. Our work identified the most deleterious mutations predicted to interfere with drug efficacy. This research provides grounds to explain the varied responses in several SARS-CoV-2 patients prescribed remdesivir and/or ritonavir/nirmatrelvir. This calls for additional molecular dynamic simulations to ascertain the stability of the drug-enzyme complex complemented with targeted clinical investigations. Personalizing the anti-SARS-CoV-2 medications based on the patient’s genetic makeup is essential to ensure drug efficacy and avoid adverse effects and treatment failures.

## Electronic supplementary material

Below is the link to the electronic supplementary material.

Supplementary Material 1

## Data Availability

The datasets generated and/or analyzed during the current study are available from the corresponding author upon reasonable request.
