# Supplementary material for: In-silico screening and analysis of missense SNPs in human CYP3A4/5 affecting drug-enzyme interactions of FDA-approved COVID-19 antiviral drugs
Source: Sci Rep. 2025 Jan 16;15:2153. doi: 10.1038/s41598-025-85595-x (PMC11739396; doi:10.1038/s41598-025-85595-x)
Supplement: Supplementary file 1 — Supplementary Material 1 [file 41598_2025_85595_MOESM1_ESM.docx]

**In-silico screening and analysis of missense SNPs in human CYP3A4/5 affecting drug-enzyme interactions of FDA-approved COVID-19 antiviral drugs**

Amro A. Abdelazim^1^, Mohamad Maged^2^, Ahmed I. Abdelmaksoud^1,3^, Sameh E. Hassanin^4*^

^1^ Department of Pharmaceutical Biotechnology, College of Biotechnology, Misr University of Science and Technology, Giza, Egypt.

^2^ Applied Biotechnology Program, School of Biotechnology, Nile University, Giza, Egypt.

^3^ Industrial Biotechnology Department, Genetic Engineering and Biotechnology Research Institute, University of Sadat City, Sadat City, Egypt.

^4^ Bioinformatics Program, School of Biotechnology, Nile University, Giza, Egypt.

^*^Corresponding author: [sibrahem@nu.edu.eg](mailto:sibrahem@nu.edu.eg)

| Table S1. The most deleterious CYP3A4 SNPs and their scoring matrices (D= Disease causing/ Deleterious/ Damaging, T=Tolerated, B=Benign, N=Neutral ). | | | | | | | | | | | | | | | |
| --- | --- | --- | --- | --- | --- | --- | --- | --- | --- | --- | --- | --- | --- | --- | --- |
| Transcript | Variant ID | MUTATIONS | sift | polyphen | CADD | revel | Meta lr | Mutation assessor | Panther | SNP&GO | PhD-SNP | SNAP | Meta-SNP | FATHMM | Out of 12 |
| ENST00000651514.1 | **rs1256858657** | **C468W** | D | D | B | D | D | high | D | D | D | D | D | T | 10 |
| ENST00000651514.1 | **rs1425544636** | **C468Y** | D | D | B | D | D | high | D | D | D | D | D | T | 10 |
| ENST00000651514.1 | **rs71583803** | **F463C** | D | D | B | D | D | medium | D | D | D | D | D | D | 10 |
| ENST00000651514.1 | **rs774168721** | **K453N** | D | D | B | D | D | high | D | D | D | D | D | T | 10 |
| ENST00000651514.1 | **rs1162067586** | **G444A** | D | D | B | D | D | medium | D | D | D | D | D | D | 10 |
| ENST00000651514.1 | **rs567089575** | **N441D** | D | D | B | D | D | high | D | D | D | D | D | T | 10 |
| ENST00000651514.1 | **rs1457998170** | **P439S** | D | D | B | D | D | high | D | D | D | D | D | T | 10 |
| ENST00000651514.1 | **rs1166211708** | **G438V** | D | D | B | D | D | high | D | D | D | D | D | D | 11 |
| ENST00000651514.1 | **rs755248651** | **G436R** | D | D | B | D | D | high | D | D | D | D | D | T | 10 |
| ENST00000651514.1 | **rs1355522141** | **P434A** | D | D | B | D | D | medium | D | D | D | D | D | D | 10 |
| ENST00000651514.1 | **rs1368745625** | **R418T** | D | D | D | D | D | high | D | D | D | D | D | D | 12 |
| ENST00000651514.1 | **rs4986909** | **P416L(CYP3A4*13)** | D | D | B | D | D | high | D | D | D | D | D | T | 10 |
| ENST00000651514.1 | **rs4986909** | **P416R** | D | D | B | D | D | high | D | D | D | D | D | T | 10 |
| ENST00000651514.1 | **rs72552797** | **P411L** | D | D | B | D | D | high | D | D | D | D | D | D | 11 |
| ENST00000651514.1 | **rs1217252102** | **P411A** | D | D | B | D | D | medium | D | D | D | D | D | D | 10 |
| ENST00000651514.1 | **rs1044764678** | **W408R** | D | D | B | D | D | high | D | D | D | D | D | T | 10 |
| ENST00000651514.1 | **rs113716682** | **L401P** | D | D | B | D | D | high | D | D | D | D | D | T | 10 |
| ENST00000651514.1 | **rs1481942841** | **P397L** | D | D | B | D | D | high | D | D | D | D | D | T | 10 |
| ENST00000651514.1 | **rs1195408117** | **L364F** | D | D | B | D | D | high | D | D | D | N | D | D | 10 |
| ENST00000651514.1 | **rs71581998** | **V359E** | D | D | B | D | D | high | D | D | D | D | D | T | 10 |
| ENST00000651514.1 | **rs754968125** | **M358V** | D | D | B | D | D | high | D | D | D | D | D | T | 10 |
| ENST00000651514.1 | **rs1201319750** | **Y355C** | D | D | B | D | D | high | D | D | D | D | D | T | 10 |
| ENST00000651514.1 | **rs1462817145** | **Y355H** | D | D | B | D | D | high | D | D | D | D | D | T | 10 |
| ENST00000651514.1 | **rs368296206** | **I335T(CYP3A4*32)** | D | D | B | D | D | high | D | D | D | D | D | T | 10 |
| ENST00000651514.1 | **rs867315029** | **E334K** | D | D | B | D | D | high | D | D | D | D | D | D | 11 |
| ENST00000651514.1 | **rs201821708** | **Y319C(CYP3A4*21)** | D | D | B | D | D | high | D | D | D | D | D | T | 10 |
| ENST00000651514.1 | **rs190354371** | **L314P** | D | D | B | D | D | high | D | D | D | D | D | T | 10 |
| ENST00000651514.1 | **rs267601666** | **L314F** | D | D | B | D | D | high | D | D | D | D | D | T | 10 |
| ENST00000651514.1 | **rs751246524** | **T310M** | D | D | B | D | D | high | D | D | D | D | D | T | 10 |
| ENST00000651514.1 | **rs375997724** | **T309I** | D | D | B | D | D | medium | D | D | D | D | D | D | 10 |
| ENST00000651514.1 | **rs71581996** | **A305S** | D | D | B | D | D | high | D | D | D | D | D | T | 10 |
| ENST00000651514.1 | **rs1368114928** | **L272P** | D | D | B | D | D | high | D | D | D | D | D | T | 10 |
| ENST00000651514.1 | **rs1166537703** | **L229R** | D | D | B | D | D | high | D | D | D | D | D | T | 10 |
| ENST00000651514.1 | **rs1467852216** | **P227L** | D | D | B | D | D | high | D | D | D | D | D | T | 10 |
| ENST00000651514.1 | **rs4987161** | **F189S(CYP3A4*17)** | D | D | B | D | D | high | D | D | D | D | D | T | 10 |
| ENST00000651514.1 | **rs773989431** | **Y179S** | D | D | B | D | D | high | D | D | D | D | D | T | 10 |
| ENST00000651514.1 | **rs1396501606** | **K173E** | D | D | B | D | D | high | D | D | D | D | D | T | 10 |
| ENST00000651514.1 | **rs1483230173** | **P135L** | D | D | B | D | D | high | D | D | D | D | D | T | 10 |
| ENST00000651514.1 | **rs72552799** | **R130P** | D | D | B | D | D | high | D | D | D | D | D | D | 11 |
| ENST00000651514.1 | **rs72552799** | **R130Q(CYP3A4*8)** | D | D | B | D | D | high | D | D | D | D | D | D | 11 |
| ENST00000651514.1 | **rs778013004** | **R130G** | D | D | B | D | D | high | D | D | D | D | D | D | 11 |
| ENST00000651514.1 | **rs1043569086** | **K127N** | D | D | B | D | D | high | D | D | D | D | D | T | 10 |
| ENST00000651514.1 | **rs142296281** | **R105W** | D | D | B | D | D | high | D | D | D | D | D | T | 10 |
| ENST00000651514.1 | **rs3091339** | **K96E** | D | D | B | D | D | high | D | D | D | D | D | T | 10 |
| ENST00000651514.1 | **rs1194211831** | **I90T** | D | D | B | D | D | high | D | D | D | D | D | T | 10 |
| ENST00000651514.1 | **rs59418896** | **Y68C** | D | D | B | D | D | high | D | D | D | D | D | T | 10 |
| ENST00000651514.1 | **rs760951972** | **P39L** | D | D | B | D | D | medium | D | D | D | D | D | D | 10 |

| Table S2. The most deleterious CYP3A5 SNPs and their scoring matrices (D= Disease causing/ Deleterious/ Damaging, T=Tolerated, B=Benign, N=Neutral ). | | | | | | | | | | | | | | | |
| --- | --- | --- | --- | --- | --- | --- | --- | --- | --- | --- | --- | --- | --- | --- | --- |
| Transcript | Variant ID | MUTATIONS | sift | polyphen | CADD | revel | Meta lr | Mutation assessor | Panther | SNP&GO | PhD-SNP | SNAP | Meta-SNP | FATHMM | Out of 12 |
| ENST00000222982.8 | **rs777196351** | **C467R** | D | D | B | D | D | high | D | D | D | D | D | T | 10 |
| ENST00000222982.8 | **rs147472467** | **A447V** | D | D | B | D | D | high | D | D | D | D | D | D | 11 |
| ENST00000222982.8 | **rs41279854** | **F446S(CYP3A5*10)** | D | D | B | D | D | high | D | D | D | D | D | T | 10 |
| ENST00000222982.8 | **rs377454308** | **G443S** | D | D | B | D | D | high | D | D | D | D | D | D | 11 |
| ENST00000222982.8 | **rs1267703650** | **I442T** | D | D | B | D | D | high | D | D | D | D | D | T | 10 |
| ENST00000222982.8 | **rs13220949** | **R439K** | D | D | B | D | D | high | D | D | D | D | D | D | 11 |
| ENST00000222982.8 | **rs1035394246** | **G435E** | D | D | B | D | D | high | D | D | D | D | D | T | 10 |
| ENST00000222982.8 | **rs780390510** | **G435R** | D | D | B | D | D | high | D | D | D | D | D | T | 10 |
| ENST00000222982.8 | **rs746993664** | **Y431N** | D | D | B | D | D | high | D | D | D | D | D | T | 10 |
| ENST00000222982.8 | **rs1474237861** | **F419L** | D | D | B | D | D | high | D | D | D | D | D | T | 10 |
| ENST00000222982.8 | **rs370299887** | **R418M** | D | D | D | D | D | high | D | D | D | D | D | D | 12 |
| ENST00000222982.8 | **rs140521496** | **P416S** | D | D | B | D | D | high | D | D | D | D | D | T | 10 |
| ENST00000222982.8 | **rs1299406057** | **P411R** | D | D | B | D | D | high | D | D | D | D | D | D | 11 |
| ENST00000222982.8 | **rs756677833** | **P411S** | D | D | B | D | D | high | D | D | D | D | D | D | 11 |
| ENST00000222982.8 | **rs1562985172** | **D404Y** | D | D | B | D | D | high | D | D | D | D | D | T | 10 |
| ENST00000222982.8 | **rs1252465240** | **L401P** | D | D | B | D | D | high | D | D | D | D | D | T | 10 |
| ENST00000222982.8 | **rs750222754** | **I388T** | D | D | B | D | D | high | D | D | D | D | D | T | 10 |
| ENST00000222982.8 | **rs756271054** | **R375G** | D | D | B | D | D | high | D | D | D | D | D | T | 10 |
| ENST00000222982.8 | **rs779306884** | **L364H** | D | D | B | D | D | high | D | D | D | D | D | D | 11 |
| ENST00000222982.8 | **rs1245832664** | **E362G** | D | D | B | D | D | high | D | D | D | D | D | D | 11 |
| ENST00000222982.8 | **rs149888520** | **Y355C** | D | D | B | D | D | high | D | D | D | D | D | T | 10 |
| ENST00000222982.8 | **rs1219950418** | **Q352P** | D | D | B | D | D | high | D | D | D | D | D | T | 10 |
| ENST00000222982.8 | **rs1363320186** | **Y347C** | D | D | B | D | D | high | D | D | D | D | D | T | 10 |
| ENST00000222982.8 | **rs990544214** | **I335T** | D | D | B | D | D | high | D | D | D | D | D | T | 10 |
| ENST00000222982.8 | **rs775816439** | **L331P** | D | D | B | D | D | high | D | D | D | D | D | T | 10 |
| ENST00000222982.8 | **rs1489670280** | **K330E** | D | D | B | D | D | high | D | D | D | D | D | T | 10 |
| ENST00000222982.8 | **rs991122268** | **S311R** | D | D | B | D | D | high | D | D | D | D | D | T | 10 |
| ENST00000222982.8 | **rs766695006** | **T309I** | D | D | B | D | D | high | D | D | D | D | D | D | 11 |
| ENST00000222982.8 | **rs766695006** | **T309N** | D | D | B | D | D | high | D | D | D | D | D | D | 11 |
| ENST00000222982.8 | **rs1562991842** | **E294K** | D | D | B | D | D | high | D | D | D | D | D | T | 10 |
| ENST00000222982.8 | **rs753396001** | **M275R** | D | D | B | D | D | high | D | D | D | D | D | T | 10 |
| ENST00000222982.8 | **rs756839053** | **L274P** | D | D | B | D | D | high | D | D | D | D | D | T | 10 |
| ENST00000222982.8 | **rs968643967** | **F248C** | D | D | B | D | D | high | D | D | D | D | D | T | 10 |
| ENST00000222982.8 | **rs979438885** | **S195F** | D | D | B | D | D | high | D | D | D | D | D | T | 10 |
| ENST00000222982.8 | **rs1215839981** | **S195P** | D | D | B | D | D | high | D | D | D | D | D | T | 10 |
| ENST00000222982.8 | **rs781765557** | **F189L** | D | D | B | D | D | high | D | D | D | D | D | T | 10 |
| ENST00000222982.8 | **rs777252753** | **V183G** | D | D | B | D | D | high | D | D | D | D | D | T | 10 |
| ENST00000222982.8 | **rs1584446515** | **D182N** | D | D | B | D | D | high | D | D | D | D | D | T | 10 |
| ENST00000222982.8 | **rs764576184** | **M145T** | D | D | B | D | D | high | D | D | D | D | D | T | 10 |
| ENST00000222982.8 | **rs764608096** | **G140R** | D | D | B | D | D | high | D | D | D | D | D | T | 10 |
| ENST00000222982.8 | **rs1383200930** | **L133P** | D | D | B | D | D | high | D | D | D | D | D | T | 10 |
| ENST00000222982.8 | **rs758037875** | **R130Q** | D | D | B | D | D | high | D | D | D | D | D | D | 11 |
| ENST00000222982.8 | **rs1294331881** | **W126R** | D | D | B | D | D | high | D | D | D | D | D | T | 10 |
| ENST00000222982.8 | **rs1186517408** | **N104I** | D | D | B | D | D | high | D | D | D | D | D | T | 10 |
| ENST00000222982.8 | **rs761882111** | **T103K** | D | D | B | D | D | high | D | D | D | D | D | T | 10 |
| ENST00000222982.8 | **rs752003788** | **L94Q** | D | D | B | D | D | high | D | D | D | D | D | T | 10 |
| ENST00000222982.8 | **rs56244447** | **L82R(CYP3A5*3D)** | D | D | B | D | D | high | D | D | D | D | D | T | 10 |

| Table S3. CYP3A4 SNPs with minor allelic frequency (Chr:bp= base pairs location on chromosome, gmaf= global minor allelic frequency, AA=amino acid, coord=coordinates/location) . SNPs that are colored red scored 10 or higher in the deleteriousness assessment and hence were considered to be most deleterious. | | | | | | | | | |
| --- | --- | --- | --- | --- | --- | --- | --- | --- | --- |
| **Variant ID** | **Location** | **Chr: bp** | **Alleles** | **gmaf_allele** | **gmaf_freq** | **Global MAF** | **AA** | **AA coord** | **SNP** |
| rs138550343 | 7:99758180 | 7:99758180 | C/T | T | 0.0002 | < 0.001 | V/I | 489 | V489I |
| rs181210913 | 7:99760827 | 7:99760827 | C/T | T | 0.0002 | < 0.001 | E/K | 470 | E470K |
| rs4986913 | 7:99760836 | 7:99760836 | G/A/C | A | 0.000399 | < 0.001 | P/S | 467 | P467S |
| rs4986913 | 7:99760836 | 7:99760836 | G/A/C | A | 0.000399 | < 0.001 | P/A | 467 | P467A |
| rs530166880 | 7:99760857 | 7:99760857 | G/A | A | 0.0002 | < 0.001 | L/F | 460 | L460F |
| rs4986910 | 7:99760901 | 7:99760901 | A/G | G | 0.002596 | 0.003 | M/T | 445 | M445T |
| rs567089575 | 7:99760914 | 7:99760914 | T/C | C | 0.0002 | < 0.001 | N/D | 441 | N441D |
| rs139109027 | 7:99760958 | 7:99760958 | T/C | C | 0.000399 | < 0.001 | N/S | 426 | N426S |
| rs142425279 | 7:99762111 | 7:99762111 | T/C | C | 0.0002 | < 0.001 | M/V | 395 | M395V |
| rs12721629 | 7:99762177 | 7:99762177 | G/A | A | 0.001198 | 0.001 | L/F | 373 | L373F |
| rs145669559 | 7:99762189 | 7:99762189 | T/C | C | 0.0002 | < 0.001 | I/V | 369 | I369V |
| rs67784355 | 7:99762206 | 7:99762206 | G/A/T | A | 0.000399 | < 0.001 | T/M | 363 | T363M |
| rs67784355 | 7:99762206 | 7:99762206 | G/A/T | A | 0.000399 | < 0.001 | T/K | 363 | T363K |
| rs201821708 | 7:99763925 | 7:99763925 | T/C | C | 0.0002 | < 0.001 | Y/C | 319 | Y319C |
| rs190354371 | 7:99763940 | 7:99763940 | A/G | G | 0.0002 | < 0.001 | L/P | 314 | L314P |
| rs201286895 | 7:99763983 | 7:99763983 | T/C | C | 0.0002 | < 0.001 | I/V | 300 | I300V |
| rs28371759 | 7:99764003 | 7:99764003 | A/G | G | 0.002196 | 0.002 | L/P | 293 | L293P |
| rs540702483 | 7:99764007 | 7:99764007 | C/T | T | 0.0002 | < 0.001 | D/N | 292 | D292N |
| rs199908125 | 7:99767145 | 7:99767145 | C/T | T | 0.0002 | < 0.001 | E/K | 262 | E262K |
| rs565580106 | 7:99768355 | 7:99768355 | T/C | C | 0.0002 | < 0.001 | I/M | 223 | I223M |
| rs55785340 | 7:99768360 | 7:99768360 | A/G/T | G | 0.000399 | < 0.001 | S/P | 222 | S222P |
| rs55785340 | 7:99768360 | 7:99768360 | A/G/T | G | 0.000399 | < 0.001 | S/T | 222 | S222T |
| rs55901263 | 7:99768371 | 7:99768371 | G/C | C | 0.000399 | < 0.001 | P/R | 218 | P218R |
| rs559410716 | 7:99768420 | 7:99768420 | G/A | A | 0.0002 | < 0.001 | P/S | 202 | P202S |
| rs113667357 | 7:99768424 | 7:99768424 | T/A/C | C | 0.000399 | < 0.001 | Q/H | 200 | Q200H |
| rs548684407 | 7:99768444 | 7:99768444 | C/A/T | T | 0.0002 | < 0.001 | D/Y | 194 | D194Y |
| rs548684407 | 7:99768444 | 7:99768444 | C/A/T | T | 0.0002 | < 0.001 | D/N | 194 | D194N |
| rs4987159 | 7:99768445 | 7:99768445 | G/A/C | A | 0.01797 | 0.018 | I/M | 193 | I193M |
| rs12721627 | 7:99768470 | 7:99768470 | G/C | C | 0.000799 | < 0.001 | T/S | 185 | T185S |
| rs571616925 | 7:99768471 | 7:99768471 | T/C | C | 0.0002 | < 0.001 | T/A | 185 | T185A |
| rs538632608 | 7:99768492 | 7:99768492 | C/A | A | 0.0002 | < 0.001 | A/S | 178 | A178S |
| rs547453529 | 7:99768501 | 7:99768501 | C/T | T | 0.0002 | < 0.001 | V/I | 175 | V175I |
| rs4986908 | 7:99769769 | 7:99769769 | C/G/T | G | 0.001797 | 0.002 | D/H | 174 | D174H |
| rs4986908 | 7:99769769 | 7:99769769 | C/G/T | G | 0.001797 | 0.002 | D/N | 174 | D174N |
| rs72552798 | 7:99769781 | 7:99769781 | C/T | T | 0.0002 | < 0.001 | V/I | 170 | V170I |
| rs568779023 | 7:99769785 | 7:99769785 | C/G/T | G | 0.000599 | < 0.001 | K/N | 168 | K168N |
| rs4986907 | 7:99769804 | 7:99769804 | C/T | T | 0.005192 | 0.005 | R/Q | 162 | R162Q |
| rs57409622 | 7:99769805 | 7:99769805 | G/A | A | 0.001198 | 0.001 | R/W | 162 | R162W |
| rs546282579 | 7:99769847 | 7:99769847 | T/C | C | 0.0002 | < 0.001 | I/V | 148 | I148V |
| rs72552799 | 7:99770165 | 7:99770165 | C/G/T | T | 0.0002 | < 0.001 | R/P | 130 | R130P |
| rs72552799 | 7:99770165 | 7:99770165 | C/G/T | T | 0.0002 | < 0.001 | R/Q | 130 | R130Q |
| rs551809042 | 7:99770169 | 7:99770169 | A/T | T | 0.0002 | < 0.001 | L/I | 129 | L129I |
| rs55951658 | 7:99770202 | 7:99770202 | T/A/C | C | 0.000998 | < 0.001 | I/F | 118 | I118F |
| rs55951658 | 7:99770202 | 7:99770202 | T/A/C | C | 0.000998 | < 0.001 | I/V | 118 | I118V |
| rs570051168 | 7:99784018 | 7:99784018 | G/C | C | 0.000799 | < 0.001 | L/V | 22 | L22V |
| rs188389063 | 7:99784075 | 7:99784075 | G/C | C | 0.0002 | < 0.001 | L/V | 3 | L3V |
| rs371360704 | 7:99784077 | 7:99784077 | G/A/C/T | T | 0.0002 | < 0.001 | A/V | 2 | A2V |
| rs371360704 | 7:99784077 | 7:99784077 | G/A/C/T | T | 0.0002 | < 0.001 | A/G | 2 | A2G |
| rs371360704 | 7:99784077 | 7:99784077 | G/A/C/T | T | 0.0002 | < 0.001 | A/D | 2 | A2D |

| Table S4. CYP3A5 SNPs with minor allelic frequency (Chr:bp= base pairs location on chromosome, gmaf= global minor allelic frequency, AA= amino acid, coord=coordinates/location). SNPs that are colored red scored 10 or higher in the deleteriousness assessment and hence were considered to be most deleterious. | | | | | | | | | |
| --- | --- | --- | --- | --- | --- | --- | --- | --- | --- |
| **Variant ID** | **Location** | **Chr: bp** | **Alleles** | **gmaf_allele** | **gmaf_freq** | **Global MAF** | **AA** | **AA coord** | **SNP** |
| rs28365085 | 7:99648351 | 7:99648351 | A/G | G | 0.001597 | 0.002 | I/T | 488 | I488T |
| rs559863537 | 7:99648381 | 7:99648381 | G/A | A | 0.0002 | < 0.001 | T/M | 478 | T478M |
| rs184177673 | 7:99648387 | 7:99648387 | A/G | G | 0.000599 | < 0.001 | L/S | 476 | L476S |
| rs41279854 | 7:99650149 | 7:99650149 | A/G | G | 0.0002 | < 0.001 | F/S | 446 | F446S |
| rs188908808 | 7:99650179 | 7:99650179 | G/A/C/T | C | 0.000998 | < 0.001 | T/I | 436 | T436I |
| rs188908808 | 7:99650179 | 7:99650179 | G/A/C/T | C | 0.000998 | < 0.001 | T/S | 436 | T436S |
| rs188908808 | 7:99650179 | 7:99650179 | G/A/C/T | C | 0.000998 | < 0.001 | T/N | 436 | T436N |
| rs28365083 | 7:99652613 | 7:99652613 | G/T | T | 0.000799 | < 0.001 | T/N | 398 | T398N |
| rs149888520 | 7:99652742 | 7:99652742 | T/C | C | 0.000599 | < 0.001 | Y/C | 355 | Y355C |
| rs149081683 | 7:99652758 | 7:99652758 | C/T | T | 0.0002 | < 0.001 | V/M | 350 | V350M |
| rs188366390 | 7:99660530 | 7:99660530 | T/A/G | G | 0.0002 | < 0.001 | Q/L | 332 | Q332L |
| rs188366390 | 7:99660530 | 7:99660530 | T/A/G | G | 0.0002 | < 0.001 | Q/P | 332 | Q332P |
| rs560280526 | 7:99660549 | 7:99660549 | C/T | T | 0.0002 | < 0.001 | D/N | 326 | D326N |
| rs527393064 | 7:99660575 | 7:99660575 | G/A/C | A | 0.0002 | < 0.001 | T/I | 317 | T317I |
| rs527393064 | 7:99660575 | 7:99660575 | G/A/C | A | 0.0002 | < 0.001 | T/S | 317 | T317S |
| rs201632515 | 7:99660585 | 7:99660585 | G/T | T | 0.0002 | < 0.001 | L/I | 314 | L314I |
| rs111371159 | 7:99662839 | 7:99662839 | G/A/T | T | 0.0002 | < 0.001 | S/L | 281 | S281L |
| rs145774441 | 7:99662854 | 7:99662854 | A/C/G | G | 0.000799 | < 0.001 | I/S | 276 | I276S |
| rs145774441 | 7:99662854 | 7:99662854 | A/C/G | G | 0.000799 | < 0.001 | I/T | 276 | I276T |
| rs542523237 | 7:99664053 | 7:99664053 | A/G/T | G | 0.0002 | < 0.001 | V/A | 238 | V238A |
| rs542523237 | 7:99664053 | 7:99664053 | A/G/T | G | 0.0002 | < 0.001 | V/D | 238 | V238D |
| rs56411402 | 7:99665237 | 7:99665237 | T/C | C | 0.000799 | < 0.001 | Q/R | 200 | Q200R |
| rs540181281 | 7:99666601 | 7:99666601 | T/C | C | 0.0002 | < 0.001 | D/G | 174 | D174G |
| rs142823108 | 7:99666676 | 7:99666676 | A/G | G | 0.000599 | < 0.001 | I/T | 149 | I149T |
| rs539204136 | 7:99666977 | 7:99666977 | G/T | T | 0.0002 | < 0.001 | T/N | 136 | T136N |
| rs189107290 | 7:99666990 | 7:99666990 | A/C/G | G | 0.0002 | < 0.001 | L/V | 132 | L132V |
| rs41279857 | 7:99672599 | 7:99672599 | G/C/T | T | 0.000599 | < 0.001 | S/C | 100 | S100C |
| rs41279857 | 7:99672599 | 7:99672599 | G/C/T | T | 0.000599 | < 0.001 | S/Y | 100 | S100Y |
| rs186648988 | 7:99676119 | 7:99676119 | C/T | T | 0.0002 | < 0.001 | R/H | 54 | R54H |
| rs566629410 | 7:99676120 | 7:99676120 | G/A/C/T | C | 0.000799 | < 0.001 | R/C | 54 | R54C |
| rs566629410 | 7:99676120 | 7:99676120 | G/A/C/T | C | 0.000799 | < 0.001 | R/G | 54 | R54G |
| rs566629410 | 7:99676120 | 7:99676120 | G/A/C/T | C | 0.000799 | < 0.001 | R/S | 54 | R54S |
| rs80026734 | 7:99676132 | 7:99676132 | C/T | T | 0.0002 | < 0.001 | V/I | 50 | V50I |
| rs28383468 | 7:99676192 | 7:99676192 | G/A | A | 0.003395 | 0.003 | H/Y | 30 | H30Y |

| Table S5. Stability determination of the mutations of CYP3A4 SNPs. | | | | | | | |
| --- | --- | --- | --- | --- | --- | --- | --- |
| Variant ID | CYP3A4 SNPs | I-Mutant | I-Mutant DDG | MU-Pro | MU-Pro DDG | INPS | INPS DDG |
| rs1256858657 | **C468W** | Decrease Stability | -0.01 | DECREASE stability | -1.0186482 | Decrease Stability | -1.39135 |
| rs1425544636 | **C468Y** | **Increase Stability** | 0.67 | DECREASE stability | -0.68608824 | Decrease Stability | -1.11001 |
| rs71583803 | **F463C** | Decrease Stability | -0.54 | DECREASE stability | -1.695776 | Decrease Stability | -2.24561 |
| rs774168721 | **K453N** | Decrease Stability | -0.04 | DECREASE stability | -0.57225128 | Decrease Stability | -0.96517 |
| rs1162067586 | **G444A** | **Increase Stability** | 0.14 | DECREASE stability | -1.0848448 | Decrease Stability | -1.18166 |
| rs567089575 | **N441D** | Decrease Stability | -1.17 | DECREASE stability | -0.87609089 | Decrease Stability | -0.474934 |
| rs1457998170 | **P439S** | Decrease Stability | -2.11 | DECREASE stability | -0.96815526 | Decrease Stability | -0.885682 |
| rs1166211708 | **G438V** | Decrease Stability | -0.66 | DECREASE stability | -0.02535065 | Decrease Stability | -1.58903 |
| rs755248651 | **P434A** | Decrease Stability | -2.61 | DECREASE stability | -2.0632659 | Decrease Stability | -0.926407 |
| rs1355522141 | **G436R** | Decrease Stability | -0.63 | DECREASE stability | -0.19317235 | Decrease Stability | -0.56599 |
| rs1368745625 | **R418T** | Decrease Stability | -0.94 | DECREASE stability | -1.277499 | Decrease Stability | -0.281713 |
| rs4986909 | **P416L(CYP3A4*13)** | **Increase Stability** | 0.2 | **INCREASE stability** | 0.08678679 | Decrease Stability | -0.964981 |
| rs4986909 | **P416R** | **Increase Stability** | 0.23 | DECREASE stability | -0.45795443 | **Increase Stability** | 0.113408 |
| rs1217252102 | **P411A** | Decrease Stability | -1.13 | DECREASE stability | -0.71771251 | Decrease Stability | -0.867728 |
| rs72552797 | **P411L** | **Increase Stability** | 0.75 | **INCREASE stability** | 0.35964056 | Decrease Stability | -1.00037 |
| rs1044764678 | **W408R** | Decrease Stability | -1.45 | DECREASE stability | -0.53866259 | Decrease Stability | -1.82756 |
| rs113716682 | **L401P** | Decrease Stability | -1.18 | DECREASE stability | -3.1763486 | Decrease Stability | -2.49155 |
| rs1481942841 | **P397L** | Decrease Stability | -0.93 | **INCREASE stability** | 0.25603015 | Decrease Stability | -0.749353 |
| rs1195408117 | **L364F** | Decrease Stability | -0.76 | DECREASE stability | -1.7931652 | Decrease Stability | -1.01474 |
| rs71581998 | **V359E** | Decrease Stability | -1.36 | DECREASE stability | -1.7643193 | Decrease Stability | -2.11742 |
| rs754968125 | **M358V** | Decrease Stability | -0.2 | DECREASE stability | -0.74978165 | Decrease Stability | -1.72138 |
| rs1201319750 | **Y355C** | **Increase Stability** | 0.55 | DECREASE stability | -0.91564367 | Decrease Stability | -1.53395 |
| rs1462817145 | **Y355H** | Decrease Stability | -0.91 | DECREASE stability | -1.3205296 | Decrease Stability | -0.977905 |
| rs368296206 | **I335T** | Decrease Stability | -3.63 | DECREASE stability | -2.0347497 | Decrease Stability | -3.14927 |
| rs867315029 | **E334K** | Decrease Stability | -0.9 | DECREASE stability | -1.4641851 | **Increase Stability** | 0.0130307 |
| rs201821708 | **Y319C** | **Increase Stability** | 1.03 | DECREASE stability | -0.15122406 | Decrease Stability | -1.53599 |
| rs190354371 | **L314P** | Decrease Stability | -1.05 | DECREASE stability | -1.9285346 | Decrease Stability | -3.1771 |
| rs267601666 | **L314F** | Decrease Stability | -0.75 | DECREASE stability | -1.1385615 | Decrease Stability | -0.912976 |
| rs751246524 | **T310M** | **Increase Stability** | 0.48 | **INCREASE stability** | 0.03593779 | Decrease Stability | -0.66729 |
| rs375997724 | **T309I** | Decrease Stability | -0.41 | DECREASE stability | -0.28272947 | Decrease Stability | -1.02607 |
| rs71581996 | **A305S** | Decrease Stability | -0.33 | DECREASE stability | -0.59017411 | Decrease Stability | -1.23116 |
| rs1368114928 | **L272P** | Decrease Stability | -1.56 | DECREASE stability | -2.3603805 | Decrease Stability | -3.12543 |
| rs1166537703 | **L229R** | Decrease Stability | -2.7 | DECREASE stability | -1.5390439 | Decrease Stability | -1.13829 |
| rs1467852216 | **P227L** | Decrease Stability | -0.7 | DECREASE stability | -0.25164685 | Decrease Stability | -1.06054 |
| rs4987161 | **F189S(CYP3A4*17)** | Decrease Stability | -3.55 | DECREASE stability | -1.6494541 | Decrease Stability | -2.9773 |
| rs773989431 | **Y179S** | Decrease Stability | -1.6 | DECREASE stability | -1.4914408 | Decrease Stability | -2.02968 |
| rs1396501606 | **K173E** | Decrease Stability | -0.39 | DECREASE stability | -0.45363835 | Decrease Stability | -0.301095 |
| rs1483230173 | **P135L** | Decrease Stability | -0.46 | DECREASE stability | -0.12983719 | Decrease Stability | -1.04178 |
| rs72552799 | **R130P** | Decrease Stability | -1.05 | DECREASE stability | -0.94981012 | Decrease Stability | -0.349157 |
| rs72552799 | **R130Q(CYP3A4*8)** | Decrease Stability | -1.09 | DECREASE stability | -0.6110723 | Decrease Stability | -0.939416 |
| rs778013004 | **R130G** | Decrease Stability | -1.24 | DECREASE stability | -1.3597011 | Decrease Stability | -0.0524399 |
| rs1043569086 | **K127N** | Decrease Stability | -1.44 | DECREASE stability | -0.64952015 | Decrease Stability | -1.00724 |
| rs142296281 | **R105W** | Decrease Stability | -0.9 | DECREASE stability | -0.67807938 | Decrease Stability | -0.797736 |
| rs3091339 | **K96E** | Decrease Stability | -0.87 | **INCREASE stability** | 0.05491429 | Decrease Stability | -0.779332 |
| rs1194211831 | **I90T** | Decrease Stability | -1.56 | DECREASE stability | -2.5672871 | Decrease Stability | -2.89611 |
| rs59418896 | **Y68C** | Decrease Stability | -0.73 | DECREASE stability | -1.4420561 | Decrease Stability | -1.53395 |
| rs760951972 | **P39L** | **Increase Stability** | 0.24 | DECREASE stability | -0.12082409 | Decrease Stability | -0.964981 |

| Table S6. Stability determination of the mutations of CYP3A5 SNPs. | | | | | | | |
| --- | --- | --- | --- | --- | --- | --- | --- |
| Variant ID | CYP3A4 SNPs | I-Mutant | I-Mutant DDG | MU-Pro | MU-Pro DDG | INPS | INPS DDG |
| rs777196351 | **C467R** | Decrease Stability | -0.53 | DECREASE stability | -1.0663619 | Decrease Stability | -0.905919 |
| rs147472467 | **A447V** | Decrease Stability | -0.07 | DECREASE stability | -1.0429684 | Decrease Stability | -1.28038 |
| rs41279854 | **F446S(CYP3A5*10)** | Decrease Stability | -2.03 | DECREASE stability | -1.461288 | Decrease Stability | -2.98575 |
| rs377454308 | **G443S** | Decrease Stability | -0.33 | DECREASE stability | -1.0684641 | Decrease Stability | -1.01657 |
| rs1267703650 | **I442T** | Decrease Stability | -2.37 | DECREASE stability | -1.6556102 | Decrease Stability | -3.01639 |
| rs13220949 | **R439K** | Decrease Stability | -1.91 | DECREASE stability | -0.57909134 | Decrease Stability | -1.40501 |
| rs1035394246 | **G435E** | Decrease Stability | -0.83 | DECREASE stability | -0.29250757 | Decrease Stability | -1.09532 |
| rs780390510 | **G435R** | Decrease Stability | -1.51 | DECREASE stability | -0.36439614 | Decrease Stability | -0.56599 |
| rs746993664 | **Y431N** | Decrease Stability | -2.71 | DECREASE stability | -1.060724 | Decrease Stability | -1.99401 |
| rs1474237861 | **F419L** | Decrease Stability | -1.46 | DECREASE stability | -0.94831532 | Decrease Stability | -2.48301 |
| rs370299887 | **R418M** | Decrease Stability | -1.48 | DECREASE stability | -0.66956479 | **Increase Stability** | 0.450277 |
| rs140521496 | **P416S** | Decrease Stability | -0.13 | DECREASE stability | -1.0478569 | Decrease Stability | -0.810742 |
| rs1299406057 | **P411R** | **Increase Stability** | 0.56 | DECREASE stability | -0.42864233 | Decrease Stability | -0.328004 |
| rs756677833 | **P411S** | Decrease Stability | -0.29 | DECREASE stability | -0.60059767 | Decrease Stability | -1.06912 |
| rs1562985172 | **D404Y** | **Increase Stability** | 1.19 | DECREASE stability | -0.96259009 | Decrease Stability | -0.208169 |
| rs1252465240 | **L401P** | Decrease Stability | -1.14 | DECREASE stability | -2.7625647 | Decrease Stability | -2.59968 |
| rs750222754 | **I388T** | Decrease Stability | -1.92 | DECREASE stability | -2.1853038 | Decrease Stability | -3.35025 |
| rs756271054 | **R375G** | Decrease Stability | -1.02 | DECREASE stability | -1.5861708 | Decrease Stability | -0.727946 |
| rs779306884 | **L364H** | Decrease Stability | -3.57 | DECREASE stability | -2.3994128 | Decrease Stability | -2.19679 |
| rs1245832664 | **E362G** | Decrease Stability | -1.2 | DECREASE stability | -1.4993466 | Decrease Stability | -0.0582868 |
| rs149888520 | **Y355C** | **Increase Stability** | 0.74 | DECREASE stability | -0.91564367 | Decrease Stability | -1.48559 |
| rs1219950418 | **Q352P** | Decrease Stability | -1.11 | DECREASE stability | -0.87130787 | Decrease Stability | -0.765601 |
| rs1363320186 | **Y347C** | **Increase Stability** | 0.47 | DECREASE stability | -1.0576138 | Decrease Stability | -1.51899 |
| rs990544214 | **I335T** | Decrease Stability | -2.84 | DECREASE stability | -2.2006156 | Decrease Stability | -3.11905 |
| rs775816439 | **L331P** | Decrease Stability | -1.45 | DECREASE stability | -2.0627388 | Decrease Stability | -3.17882 |
| rs1489670280 | **K330E** | **Increase Stability** | 0.1 | **INCREASE stability** | 0.32837513 | Decrease Stability | -0.842402 |
| rs991122268 | **S311R** | Decrease Stability | -0.9 | DECREASE stability | -0.29350264 | Decrease Stability | -0.25852 |
| rs766695006 | **T309I** | Decrease Stability | -0.26 | DECREASE stability | -0.28272947 | Decrease Stability | -1.05006 |
| rs766695006 | **T309N** | Decrease Stability | -1.08 | DECREASE stability | -0.9033886 | Decrease Stability | -0.924229 |
| rs1562991842 | **E294K** | Decrease Stability | -0.81 | DECREASE stability | -1.3346357 | Decrease Stability | -0.67912 |
| rs753396001 | **M275R** | Decrease Stability | -0.48 | DECREASE stability | -1.5452833 | Decrease Stability | -1.01951 |
| rs756839053 | **L274P** | Decrease Stability | -0.73 | DECREASE stability | -1.849939 | Decrease Stability | -2.59968 |
| rs968643967 | **F248C** | Decrease Stability | -2.39 | DECREASE stability | -1.5571209 | Decrease Stability | -1.80163 |
| rs979438885 | **S195F** | Decrease Stability | -0.28 | DECREASE stability | -0.46947696 | **Increase Stability** | 0.401828 |
| rs1215839981 | **S195P** | Decrease Stability | -2.33 | DECREASE stability | -1.3218521 | Decrease Stability | -0.810768 |
| rs781765557 | **F189L** | Decrease Stability | -3.39 | DECREASE stability | -0.65608281 | Decrease Stability | -1.75108 |
| rs777252753 | **V183G** | Decrease Stability | -4.87 | DECREASE stability | -2.756699 | Decrease Stability | -3.65817 |
| rs1584446515 | **D182N** | Decrease Stability | -2.13 | DECREASE stability | -1.1268921 | Decrease Stability | -0.422282 |
| rs764576184 | **M145T** | Decrease Stability | -0.82 | DECREASE stability | -2.0392803 | Decrease Stability | -2.1169 |
| rs764608096 | **G140R** | Decrease Stability | -1.74 | DECREASE stability | -0.83616425 | Decrease Stability | 0.928759 |
| rs1383200930 | **L133P** | Decrease Stability | -0.32 | DECREASE stability | -1.7296289 | Decrease Stability | -3.17061 |
| rs758037875 | **R130Q** | Decrease Stability | -1.39 | DECREASE stability | -0.81620029 | Decrease Stability | -0.939416 |
| rs1294331881 | **W126R** | Decrease Stability | -1.38 | DECREASE stability | -1.3278804 | Decrease Stability | -1.86392 |
| rs1186517408 | **N104I** | **Increase Stability** | 0.65 | DECREASE stability | -0.66890115 | Decrease Stability | 0.180287 |
| rs761882111 | **T103K** | Decrease Stability | -1.27 | DECREASE stability | -1.4424859 | Decrease Stability | -0.25518 |
| rs752003788 | **L94Q** | Decrease Stability | -2.27 | DECREASE stability | -1.5528422 | Decrease Stability | -2.00178 |
| rs56244447 | **L82R (CYP3A5*3D)** | Decrease Stability | -3.1 | DECREASE stability | -1.533885 | Decrease Stability | -1.37796 |

| Table S7. Consurf Conservation analysis results of CYP3A4. | | | |  |
| --- | --- | --- | --- | --- |
| CYP3A4 amino acids | Consurf_score | Exposed / Buried | Structural / Functional | |
| C468 | **6** | **Exposed** | **N/A** | |
| F463 | **6** | **Buried** | **N/A** | |
| K453 | **9** | **Buried** | **Structural** | |
| G444 | **9** | **Buried** | **Structural** | |
| N441 | **8** | **Exposed** | **Functional** | |
| P439 | **9** | **Exposed** | **Functional** | |
| G438 | **9** | **Exposed** | **Functional** | |
| G436 | **9** | **Exposed** | **Functional** | |
| P434 | **9** | **Buried** | **Structural** | |
| R418 | **9** | **Buried** | **Structural** | |
| P416 | **9** | **Buried** | **Structural** | |
| P411 | **9** | **Buried** | **Structural** | |
| W408 | **9** | **Buried** | **N/A** | |
| L401 | **7** | **Buried** | **N/A** | |
| P397 | **7** | **Buried** | **N/A** | |
| L364 | **8** | **Buried** | **N/A** | |
| V359 | **8** | **Buried** | **N/A** | |
| M358 | **8** | **Buried** | **N/A** | |
| Y355 | **9** | **Buried** | **Structural** | |
| I335 | **8** | **Buried** | **N/A** | |
| E334 | **9** | **Exposed** | **Functional** | |
| Y319 | **8** | **Buried** | **N/A** | |
| L314 | **8** | **Buried** | **N/A** | |
| T310 | **8** | **Buried** | **N/A** | |
| T309 | **9** | **Exposed** | **Functional** | |
| A305 | **8** | **Exposed** | **Functional** | |
| L272 | **8** | **Buried** | **N/A** | |
| L229 | **5** | **Exposed** | **N/A** | |
| P227 | **9** | **Exposed** | **Functional** | |
| F189 | **9** | **Buried** | **Structural** | |
| Y179 | **7** | **Buried** | **N/A** | |
| K173 | **7** | **Exposed** | **N/A** | |
| P135 | **9** | **Exposed** | **Functional** | |
| R130 | **9** | **Exposed** | **Functional** | |
| K127 | **8** | **Exposed** | **Functional** | |
| R105 | **9** | **Exposed** | **Functional** | |
| K96 | **9** | **Exposed** | **Functional** | |
| I90 | **8** | **Buried** | **N/A** | |
| Y68 | **8** | **Exposed** | **N/A** | |
| P39 | **5** | **Exposed** | **N/A** | |

| Table S8. Consurf Conservation analysis results of CYP3A5. | | | |
| --- | --- | --- | --- |
| CYP3A5 amino acids | Consurf_score | Exposed / Buried | Structural / Functional |
| C467 | **7** | **Exposed** | **N/A** |
| A447 | **9** | **Buried** | **Structural** |
| F446 | **8** | **Buried** | **N/A** |
| G443 | **9** | **Buried** | **Structural** |
| I442 | **8** | **Exposed** | **Functional** |
| R439 | **9** | **Buried** | **Structural** |
| G435 | **9** | **Buried** | **Structural** |
| Y431 | **6** | **Buried** | **N/A** |
| F419 | **9** | **Exposed** | **Functional** |
| R418 | **9** | **Buried** | **Structural** |
| P416 | **9** | **Buried** | **Structural** |
| P411 | **9** | **Buried** | **Structural** |
| D404 | **8** | **Buried** | **N/A** |
| L401 | **7** | **Buried** | **N/A** |
| I388 | **9** | **Buried** | **Structural** |
| R375 | **9** | **Buried** | **Structural** |
| L364 | **8** | **Buried** | **N/A** |
| E362 | **9** | **Buried** | **Structural** |
| Y355 | **9** | **Buried** | **Structural** |
| Q352 | **6** | **Exposed** | **N/A** |
| Y347 | **9** | **Buried** | **Structural** |
| I335 | **9** | **Buried** | **Structural** |
| L331 | **8** | **Buried** | **N/A** |
| K330 | **7** | **Exposed** | **N/A** |
| S311 | **8** | **Buried** | **N/A** |
| T309 | **9** | **Exposed** | **Functional** |
| T309 | **9** | **Exposed** | **Functional** |
| E294 | **8** | **Buried** | **N/A** |
| M275 | **9** | **Buried** | **Structural** |
| L274 | **7** | **Buried** | **N/A** |
| F248 | **7** | **Exposed** | **N/A** |
| S195 | **8** | **Buried** | **N/A** |
| F189 | **9** | **Buried** | **Structural** |
| V183 | **8** | **Buried** | **N/A** |
| D182 | **9** | **Buried** | **Structural** |
| M145 | **9** | **Buried** | **Structural** |
| G140 | **7** | **Exposed** | **N/A** |
| L133 | **7** | **Buried** | **N/A** |
| R130 | **9** | **Exposed** | **Functional** |
| W126 | **9** | **Buried** | **Structural** |
| N104 | **9** | **Buried** | **Structural** |
| T103 | **6** | **Buried** | **N/A** |
| L94 | **6** | **Buried** | **N/A** |
| L82 | **6** | **Buried** | **N/A** |
|  |  |  |  |

| Table S9. CYP3A4 Posttranslational modification prediction using NetPhos3.1 tool. | | |
| --- | --- | --- |
| Amino acid | Context | Score |
| 119 S | KSAISIAED | 0.993 |
| 134 S | RSLLSPTFT | 0.932 |
| 166 T | REAETGKPV | 0.964 |
| 259 S | RMKESRLED | 0.927 |
| 264 T | RLEDTQKHR | 0.919 |
| 281 S | DSQNSKETE | 0.988 |
| 286 S | KETESHKAL | 0.993 |
| 291 S | HKALSDLEL | 0.986 |
| 407 Y | RDPKYWTEP | 0.965 |

| Table S10. CYP3A5 Posttranslational modification prediction using NetPhos3.1 tool. | | |
| --- | --- | --- |
| Amino acid | Context | Score |
| 119 S | **KSAISLAED** | **0.991** |
| 134 S | **RSLLSPTFT** | **0.990** |
| 281 S | **DSQNSKETE** | **0.988** |
| 286 S | **KETESHKAL** | **0.993** |
| 291 S | **HKALSDLEL** | **0.981** |
| 407 Y | **HDPKYWTEP** | **0.943** |
| 420 S | **PERFSKKKD** | **0.997** |
| 425 S | **KKKDSIDPY** | **0.953** |
| 429 Y | **SIDPYIYTP** | **0.916** |
| 500 S | **DGTLSGE--** | **0.948** |

| Table S11. COACH Residue identifier for CYP3A4, Some of the SNPs under investigation occur in the amino acids within the enzyme's binding site and are highlighted in red. | | | |
| --- | --- | --- | --- |
| COACH Results | | | |
| C-score | **Cluster size** | **Ligand Name** | **Consensus Binding Residues** |
| 1 | 1067 | HEM | 105,118,119,126,130,302,305,306,309,310,313,364,369,370,373,375,434,435,436,440,441,442,443,444,447,448,452 |
| 0.16 | 89 | HC9 | 119,215,301,305,309,370,371,372,374,481,482 |
| 0.16 | 105 | HEM | 104,119,126,305,306,309,369,370,373,375,398,434,435,440,442,443,444,448 |
| 0.14 | 75 | 0T3 | 111,119,215,301,304,305,309,482 |
| 0.07 | 45 | 6AW | 53,57,76,106,108,119,224,301,304,305,309,370,372,374 |
| 0.06 | 63 | 3QZ | 119,120,208,211,212,215,301,304,305,308,309,369,370 |
| 0.03 | 17 | Z8Z | 76,105,106,108,119,120,211,215,224,241,301,304,305,309 |
| 0.03 | 37 | HC9 | 119,301,305,309,370,371,372,374,482 |
| 0.03 | 38 | PN0 | 119,120,214,215,242,300,301,304,305,308,309 |
| 0.02 | 28 | VFV | 107,109,118,130,224,298,301,305,370,372,374 |
| TM-SITE Results | | | |
| C-score | **Cluster size** | **Ligand Name** | **Consensus Binding Residues** |
| 0.56 | 615 | HEM (609) ,HEC (4) ,PP9 (2) | 94,118,119,126,130,137,184,305,306,309,310,313,364,369,370,375,398,434,435,436,437,440,442,443,444,447,448 |
| 0.23 | 13 | 0T3 (5) ,SYN (4) ,SRO (4) | 119,120,212,301,304,305,308,309,370 |
| 0.22 | 13 | 1YN (5) ,VNT (4) ,KLN (4) | 119,215,301,305,309,370,371,372,374,481,482 |
| 0.15 | 5 | PN0 (2) ,0T3 (2) ,RTZ (1) | 111,119,120,211,214,215,242,301,304,305,308,309,482 |
| 0.15 | 5 | 1C6 (3) ,REA (1) ,25S (1) | 53,106,119,216,304,305,309,370,371,372,397,482 |
| S-SITE Results | | | |
| C-score | **Cluster size** | **Ligand Name** | **Consensus Binding Residues** |
| 0.40 | 272 | HEM (161) ,UUU (102) , HEC (4) | 101,104,107,110,114,118,119,126,130,137,184,301,302,304,305,306,309,310,313,364,369,370,373,375,398,434, 435,436,440,441,442,443,444,447,448,481 |
| 0.22 | 112 | TB2 (8) ,140 (7) ,1CI (5) | 53,58,61,79,83,104,105,106,107,110,113,114,119,120,211,214,215,218,225,242,301,304,305,308,309,370,371,372, 373,374,481,482 |
| 0.11 | 6 | III (6) | 95,127,131,134,135,137,138,139,350,351,357,361,424,430,432,433,437,438,439,441,445,446 |
| 0.11 | 6 | CM5 (4) ,DBE (1) ,ESL (1) | 131,132,133,187,271,272,275,278,281,282,290,291,295,298,302,443 |
| 0.10 | 4 | CM5 (3) ,LNP (1) | 104,107,114,118,119,120,122,123,126,127,130,133,233,235,295,298,301,305,370 |

| Table S12. COACH Residue identifier for CYP3A5, Some of the SNPs under investigation occur in the amino acids within the enzyme's binding site and are highlighted in red. | | | |
| --- | --- | --- | --- |
| COACH Results | | | |
| C-score | **Cluster size** | **Ligand Name** | **Consensus Binding Residues** |
| 0.98 | 1135 | HEM | 104,118,119,126,130,302,305,306,309,310,313,364,369,370,373,375,433,434,435,439,440,441,442,443,446,447,451 |
| 0.16 | 104 | N4E | 119,120,301,304,305,309,370 |
| 0.10 | 67 | RIT | 105,119,120,211,241,301,304,305,309,369,370,372,373,374,480 |
| 0.09 | 84 | HC9 | 119,215,301,305,309,370,371,372,374,480,481 |
| 0.09 | 61 | 6AW | 53,57,76,106,119,224,301,304,305,309,370,372,374 |
| 0.05 | 51 | 3QZ | 119,120,211,214,215,218,301,304,305,308,309,369,370 |
| 0.04 | 68 | AER | 119,120,210,211,214,215,300,301,304,305,309 |
| 0.02 | 37 | LFS | 106,118,120,129,132,301,304,305 |
| 0.01 | 9 | 7AW | 76,106,211,212,224,241,301,305,309,369,480 |
| 0.01 | 15 | 1C6 | 57,83,119,304,305,370,371,372 |
| TM-SITE Results | | | |
| C-score | **Cluster size** | **Ligand Name** | **Consensus Binding Residues** |
| 0.56 | 620 | HEM (614) ,HEC (4) ,PP9 (2) | 94,118,119,126,130,137,184,305,306,309,310,313,364,369,370,373,375,398,433,434,435,436,439,441,442,443,446,447 |
| 0.24 | 13 | 0T3 (5) ,SRO (4) ,TPF (4) | 119,120,212,301,304,305,308,309,370 |
| 0.22 | 14 | 1YN(5) ,1CA(5) ,7AW (4) | 119,215,301,305,309,370,371,372,374,480,481 |
| 0.19 | 10 | PN0(4) ,AER(3) ,RTZ (3) | 119,120,211,214,215,245,300,301,304,305,308,309,369,370 |
| 0.15 | 4 | CPS(2) ,EL3(1) ,PIM (1) | 216,304,305,308,309,369,370,371,372,480 |
| S-SITE Results | | | |
| C-score | **Cluster size** | **Ligand Name** | **Consensus Binding Residues** |
| 0.40 | 271 | HEM (161), UUU (102), HEC (4) | 101,104,107,110,114,118,119,126,130,137,184,301,302,304,305,306,309,310,313,364,369,370,373,375,398,433, 434,435,439,440,441,442,443,446,447,480 |
| 0.20 | 89 | TB2 (10), HC9 (5), NCT (5) | 104,106,107,110,113,114,119,120,210,211,214,215,218,242,245,301,304,305,308,309,370,371,372,373,374,480, 481 |
| 0.12 | 26 | 140 (7) ,PLM (5) ,PAM (4) | 53,57,58,61,74,76,79,83,104,105,106,107,110,114,119,120,213,218,221,224,225,301,304,305,370,371,372,374,480,481 |
| 0.11 | 6 | III (6) | 95,127,131,134,135,137,138,139,350,351,357,361,424,429,431,432,436,437,438,440,444,445 |
| 0.11 | 6 | CM5 (4) ,ESL (1) ,DBE (1) | 131,132,133,187,271,272,275,278,281,282,290,291,295,298,302,442 |

| Table S13. SWISS-MODEL CYP3A4 models validation. | | | | | | | | | | | | | | | | |
| --- | --- | --- | --- | --- | --- | --- | --- | --- | --- | --- | --- | --- | --- | --- | --- | --- |
| Variant ID | MUTATIONS | RMSD | TM Score | Seq.Identity | Qmean | C-Beta(Cβ) | All Atoms | Solvation | Torsion | GMQE | QMEANDisCo Global | Template | MolProbity Score | Clash Score | Ramachandran Favoured | Ramachandran Outliers |
| rs1256858657 | **C468W** | **0.11** | **0.9998** | **99.78** | **0.71** | **-0.89** | **0.62** | **1.23** | **0.48** | **0.85** | **0.86** | **3nxu** | **1.89** | **2.69** | **95.92%** | **0.21%** |
| rs1425544636 | **C468Y** | **0.12** | **0.99975** | **99.78** | **0.57** | **-0.84** | **0.59** | **1.30** | **0.3** | **0.85** | **0.85** | **3nxu** | **1.89** | **2.69** | **96.14%** | **0.21%** |
| rs71583803 | **F463C** | **0.09** | **0.99987** | **99.78** | **0.63** | **-0.80** | **0.36** | **1.11** | **0.42** | **0.85** | **0.85** | **3nxu** | **1.95** | **2.82** | **95.71%** | **0.00%** |
| rs774168721 | **K453N** | **0.09** | **0.99987** | **99.78** | **0.74** | **-0.87** | **0.46** | **1.28** | **0.49** | **0.85** | **0.85** | **3nxu** | **1.93** | **2.82** | **95.92%** | **0.00%** |
| rs1162067586 | **G444A** | **0.09** | **0.99987** | **99.78** | **0.75** | **-0.80** | **0.51** | **1.24** | **0.50** | **0.85** | **0.85** | **3nxu** | **1.80** | **1.68** | **95.71%** | **0.00%** |
| rs567089575 | **N441D** | **0.09** | **0.99987** | **99.78** | **0.76** | **-0.90** | **0.48** | **1.15** | **0.56** | **0.85** | **0.85** | **3nxu** | **1.80** | **1.68** | **95.71%** | **0.21%** |
| rs1457998170 | **P439S** | **0.09** | **0.99987** | **99.78** | **0.65** | **-0.88** | **0.41** | **1.10** | **0.47** | **0.85** | **0.85** | **3nxu** | **1.95** | **2.82** | **95.71%** | **0.00%** |
| rs1166211708 | **G438V** | **0.10** | **0.99983** | **99.78** | **0.22** | **-0.86** | **0.40** | **1.09** | **0.01** | **0.85** | **0.85** | **3nxu** | **2.13** | **4.10** | **95.06%** | **0.64%** |
| rs755248651 | **G436R** | **0.09** | **0.99987** | **99.78** | **0.56** | **-0.75** | **0.49** | **1.20** | **0.31** | **0.85** | **0.85** | **3nxu** | **1.70** | **1.05** | **95.49%** | **0.21%** |
| rs1355522141 | **P434A** | **0.09** | **0.99987** | **99.78** | **0.55** | **-0.78** | **0.45** | **1.18** | **0.31** | **0.85** | **0.85** | **3nxu** | **1.82** | **1.68** | **95.49%** | **0.00%** |
| rs1368745625 | **R418T** | **0.09** | **0.99987** | **99.78** | **0.78** | **-0.92** | **0.43** | **1.37** | **0.52** | **0.85** | **0.85** | **3nxu** | **1.94** | **2.82** | **95.71%** | **0.00%** |
| rs4986909 | **P416L (CYP3A4*13)** | **0.10** | **0.99984** | **99.78** | **0.83** | **-0.91** | **0.54** | **1.27** | **0.59** | **0.85** | **0.86** | **3nxu** | **2.02** | **3.33** | **95.71%** | **0.00%** |
| rs4986909 | **P416R** | **0.09** | **0.99985** | **99.78** | **0.72** | **-0.86** | **0.36** | **1.03** | **0.56** | **0.85** | **0.86** | **3nxu** | **2.04** | **3.59** | **95.71%** | **0.00%** |
| rs72552797 | **P411L** | **0.09** | **0.99986** | **99.78** | **0.61** | **-0.88** | **0.45** | **1.23** | **0.38** | **0.85** | **0.85** | **3nxu** | **2.05** | **3.72** | **95.71%** | **0.00%** |
| rs1217252102 | **P411A** | **0.09** | **0.99987** | **99.78** | **0.69** | **-0.92** | **0.49** | **1.26** | **0.46** | **0.85** | **0.86** | **3nxu** | **1.95** | **2.82** | **95.71%** | **0.00%** |
| rs1044764678 | **W408R** | **0.09** | **0.99987** | **99.78** | **0.75** | **-0.78** | **0.35** | **1.22** | **0.51** | **0.85** | **0.86** | **3nxu** | **1.95** | **2.82** | **95.71%** | **0.00%** |
| rs113716682 | **L401P** | **0.09** | **0.99986** | **99.78** | **0.92** | **-0.84** | **0.44** | **1.22** | **0.70** | **0.85** | **0.86** | **3nxu** | **2.02** | **3.08** | **95.06%** | **0.21%** |
| rs1481942841 | **P397L** | **0.13** | **0.99973** | **99.78** | **0.80** | **-0.39** | **0.65** | **1.36** | **0.44** | **0.85** | **0.85** | **3nxu** | **1.90** | **3.08** | **96.35%** | **0.21%** |
| rs1195408117 | **L364F** | **0.10** | **0.99983** | **99.78** | **0.78** | **-0.78** | **0.51** | **1.35** | **0.50** | **0.85** | **0.85** | **3nxu** | **2.02** | **3.33** | **95.71%** | **0.21%** |
| rs71581998 | **V359E** | **0.09** | **0.99987** | **99.78** | **0.67** | **-0.92** | **0.34** | **1.08** | **0.50** | **0.85** | **0.86** | **3nxu** | **1.95** | **2.82** | **95.71%** | **0.00%** |
| rs754968125 | **M358V** | **0.10** | **0.99983** | **99.78** | **0.79** | **-0.95** | **0.43** | **1.21** | **0.58** | **0.85** | **0.85** | **3nxu** | **1.95** | **2.57** | **95.71%** | **0.21%** |
| rs1201319750 | **Y355C** | **0.09** | **0.99987** | **99.78** | **0.73** | **-0.78** | **0.40** | **1.25** | **0.48** | **0.85** | **0.85** | **3nxu** | **1.95** | **2.82** | **95.71%** | **0.00%** |
| rs1462817145 | **Y355H** | **0.09** | **0.99987** | **99.78** | **0.70** | **-0.83** | **0.40** | **1.18** | **0.48** | **0.85** | **0.85** | **3nxu** | **1.95** | **2.82** | **95.71%** | **0.00%** |
| rs368296206 | **I335T (CYP3A4*32)** | **0.09** | **0.99987** | **99.78** | **0.68** | **-0.94** | **0.38** | **1.25** | **0.46** | **0.85** | **0.85** | **3nxu** | **1.95** | **2.82** | **95.71%** | **0.00%** |
| rs867315029 | **E334K** | **0.09** | **0.99987** | **99.78** | **0.76** | **-0.73** | **0.46** | **1.23** | **0.51** | **0.85** | **0.86** | **3nxu** | **1.93** | **2.82** | **95.92%** | **0.00%** |
| rs201821708 | **Y319C (CYP3A4*21)** | **0.09** | **0.99987** | **99.78** | **0.69** | **-0.93** | **0.36** | **1.19** | **0.48** | **0.85** | **0.86** | **3nxu** | **1.95** | **2.82** | **95.71%** | **0.00%** |
| rs190354371 | **L314P** | **0.09** | **0.99986** | **99.78** | **0.68** | **-0.79** | **0.37** | **1.20** | **0.45** | **0.85** | **0.85** | **3nxu** | **1.98** | **3.08** | **95.71%** | **0.00%** |
| rs267601666 | **L314F** | **0.10** | **0.99983** | **99.78** | **0.82** | **-0.76** | **0.40** | **1.22** | **0.57** | **0.85** | **0.85** | **3nxu** | **1.93** | **2.56** | **95.71%** | **0.00%** |
| rs751246524 | **T310M** | **0.09** | **0.99987** | **99.78** | **0.81** | **-0.80** | **0.43** | **1.28** | **0.55** | **0.85** | **0.85** | **3nxu** | **1.95** | **2.82** | **95.71%** | **0.00%** |
| rs375997724 | **T309I** | **0.09** | **0.99987** | **99.78** | **0.76** | **-0.88** | **0.39** | **1.24** | **0.53** | **0.85** | **0.85** | **3nxu** | **1.63** | **0.79** | **95.71%** | **0.00%** |
| rs71581996 | **A305S** | **0.09** | **0.99987** | **99.78** | **0.70** | **-0.80** | **0.48** | **1.23** | **0.45** | **0.85** | **0.85** | **3nxu** | **1.63** | **0.79** | **95.71%** | **0.00%** |
| rs1368114928 | **L272P** | **0.10** | **0.99984** | **99.78** | **0.77** | **-1.07** | **0.34** | **1.25** | **0.58** | **0.85** | **0.85** | **3nxu** | **1.94** | **2.57** | **95.49%** | **0.00%** |
| rs1166537703 | **L229R** | **0.09** | **0.99987** | **99.78** | **0.73** | **-0.84** | **0.42** | **1.25** | **0.49** | **0.85** | **0.86** | **3nxu** | **1.94** | **2.82** | **95.71%** | **0.00%** |
| rs1467852216 | **P227L** | **0.09** | **0.99987** | **99.78** | **0.59** | **-0.79** | **0.39** | **1.14** | **0.37** | **0.85** | **0.86** | **3nxu** | **1.95** | **2.82** | **95.71%** | **0.00%** |
| rs4987161 | **F189S (CYP3A4*17)** | **0.09** | **0.99987** | **99.78** | **0.68** | **-0.94** | **0.43** | **1.25** | **0.46** | **0.85** | **0.86** | **3nxu** | **1.95** | **2.82** | **95.71%** | **0.00%** |
| rs773989431 | **Y179S** | **0.09** | **0.99987** | **99.78** | **0.68** | **-0.82** | **0.46** | **1.19** | **0.45** | **0.85** | **0.85** | **3nxu** | **1.95** | **2.82** | **95.71%** | **0.00%** |
| rs1396501606 | **K173E** | **0.09** | **0.99987** | **99.78** | **0.73** | **-0.87** | **0.40** | **1.31** | **0.47** | **0.85** | **0.85** | **3nxu** | **1.95** | **2.82** | **95.71%** | **0.00%** |
| rs1483230173 | **P135L** | **0.09** | **0.99987** | **99.78** | **0.78** | **-0.62** | **0.45** | **1.30** | **0.48** | **0.85** | **0.85** | **3nxu** | **1.95** | **2.82** | **95.71%** | **0.00%** |
| rs72552799 | **R130P** | **0.09** | **0.99985** | **99.78** | **0.81** | **-0.77** | **0.44** | **1.36** | **0.52** | **0.85** | **0.85** | **3nxu** | **1.85** | **1.94** | **95.71%** | **0.00%** |
| rs72552799 | **R130Q (CYP3A4*8)** | **0.09** | **0.99987** | **99.78** | **0.78** | **-0.78** | **0.51** | **1.39** | **0.48** | **0.85** | **0.86** | **3nxu** | **1.80** | **1.68** | **95.71%** | **0.21%** |
| rs778013004 | **R130G** | **0.09** | **0.99987** | **99.78** | **0.71** | **-0.78** | **0.50** | **1.30** | **0.43** | **0.85** | **0.86** | **3nxu** | **1.80** | **1.69** | **95.71%** | **0.21%** |
| rs1043569086 | **K127N** | **0.09** | **0.99987** | **99.78** | **0.73** | **-0.78** | **0.49** | **1.31** | **0.45** | **0.85** | **0.85** | **3nxu** | **1.96** | **2.95** | **95.71%** | **0.00%** |
| rs142296281 | **R105W** | **0.09** | **0.99987** | **99.78** | **0.75** | **-0.82** | **0.45** | **1.27** | **0.50** | **0.85** | **0.85** | **3nxu** | **1.61** | **0.79** | **95.71%** | **0.00%** |
| rs3091339 | **K96E** | **0.09** | **0.99987** | **99.78** | **0.75** | **-0.70** | **0.45** | **1.25** | **0.48** | **0.85** | **0.85** | **3nxu** | **1.95** | **2.82** | **95.71%** | **0.00%** |
| rs1194211831 | **I90T** | **0.09** | **0.99987** | **99.78** | **0.77** | **-0.73** | **0.50** | **1.34** | **0.48** | **0.85** | **0.85** | **3nxu** | **1.95** | **2.82** | **95.71%** | **0.00%** |
| rs59418896 | **Y68C** | **0.09** | **0.99987** | **99.78** | **0.76** | **-0.69** | **0.58** | **1.31** | **0.46** | **0.85** | **0.85** | **3nxu** | **1.95** | **2.82** | **95.71%** | **0.00%** |
| rs760951972 | **P39L** | **0.09** | **0.99987** | **99.78** | **0.68** | **-0.86** | **0.52** | **1.31** | **0.42** | **0.85** | **0.85** | **3nxu** | **1.95** | **2.82** | **95.71%** | **0.00%** |

| Table S14. SWISS-MODEL CYP3A5 models validation. | | | | | | | | | | | | | | | | |
| --- | --- | --- | --- | --- | --- | --- | --- | --- | --- | --- | --- | --- | --- | --- | --- | --- |
| Variant ID | MUTATIONS | RMSD | TM Score | Seq.Identity | Qmean | C-Beta(Cβ) | All Atoms | Solvation | Torsion | GMQE | QMEANDisCo Global | Template | MolProbity Score | Clash Score | Ramachandran Favoured | Ramachandran Outliers |
| rs777196351 | **C467R** | **0.06** | **0.99995** | **99.78** | **0.80** | **-0.16** | **0.51** | **1.60** | **0.31** | **0.83** | **0.83** | **5veu** | **1.20** | **1.79** | **96.15%** | **0.00%** |
| rs147472467 | **A447V** | **0.05** | **0.99995** | **99.78** | **0.87** | **0.03** | **0.59** | **1.62** | **0.35** | **0.83** | **0.83** | **5veu** | **1.04** | **1.03** | **96.36%** | **0.00%** |
| rs41279854 | **F446S (CYP3A5*10)** | **0.05** | **0.99995** | **99.78** | **0.85** | **-0.09** | **0.54** | **1.55** | **0.37** | **0.83** | **0.83** | **5veu** | **1.05** | **1.03** | **96.36%** | **0.00%** |
| rs377454308 | **G443S** | **0.05** | **0.99995** | **99.78** | **0.83** | **-0.20** | **0.47** | **1.51** | **0.38** | **0.84** | **0.83** | **5veu** | **1.27** | **2.43** | **96.36%** | **0.00%** |
| rs1267703650 | **I442T** | **0.05** | **0.99995** | **99.78** | **0.81** | **-0.23** | **0.53** | **1.54** | **0.36** | **0.83** | **0.83** | **5veu** | **1.05** | **1.03** | **96.36%** | **0.00%** |
| rs13220949 | **R439K** | **0.06** | **0.99995** | **99.78** | **0.83** | **-0.03** | **0.55** | **1.52** | **0.35** | **0.83** | **0.83** | **5veu** | **1.02** | **1.03** | **96.57%** | **0.00%** |
| rs1035394246 | **G435E** | **0.06** | **0.99995** | **99.78** | **0.54** | **-0.27** | **0.45** | **1.40** | **0.14** | **0.83** | **0.83** | **5veu** | **1.16** | **1.68** | **96.36%** | **0.00%** |
| rs780390510 | **G435R** | **0.06** | **0.99995** | **99.78** | **0.61** | **-0.25** | **0.52** | **1.51** | **0.17** | **0.83** | **0.83** | **5veu** | **1.18** | **1.80** | **96.36%** | **0.21%** |
| rs746993664 | **Y431N** | **0.05** | **0.99995** | **99.78** | **0.89** | **-0.09** | **0.54** | **1.54** | **0.41** | **0.83** | **0.83** | **5veu** | **1.14** | **1.54** | **96.36%** | **0.00%** |
| rs1474237861 | **F419L** | **0.05** | **0.99995** | **99.78** | **0.82** | **-0.28** | **0.49** | **1.58** | **0.37** | **0.84** | **0.83** | **5veu** | **1.12** | **1.41** | **96.36%** | **0.00%** |
| rs370299887 | **R418M** | **0.05** | **0.99995** | **99.78** | **0.72** | **-0.14** | **0.45** | **1.52** | **0.26** | **0.84** | **0.83** | **5veu** | **1.14** | **1.41** | **96.15%** | **0.00%** |
| rs140521496 | **P416S** | **0.05** | **0.99995** | **99.78** | **0.72** | **-0.22** | **0.46** | **1.53** | **0.26** | **0.83** | **0.83** | **5veu** | **1.12** | **1.54** | **96.57%** | **0.00%** |
| rs1299406057 | **P411R** | **0.06** | **0.99994** | **99.78** | **0.69** | **-0.14** | **0.54** | **1.49** | **0.23** | **0.83** | **0.83** | **5veu** | **1.14** | **1.53** | **96.36%** | **0.00%** |
| rs756677833 | **P411S** | **0.06** | **0.99995** | **99.78** | **0.68** | **-0.18** | **0.49** | **1.44** | **0.25** | **0.83** | **0.83** | **5veu** | **1.14** | **1.54** | **96.36%** | **0.00%** |
| rs1562985172 | **D404Y** | **0.12** | **0.99976** | **99.78** | **0.01** | **-0.22** | **0.72** | **1.40** | **-0.43** | **0.83** | **0.83** | **5veu** | **1.14** | **1.41** | **96.15%** | **0.00%** |
| rs1252465240 | **L401P** | **0.07** | **0.99991** | **99.78** | **0.71** | **0.06** | **0.54** | **1.42** | **0.24** | **0.83** | **0.83** | **5veu** | **1.12** | **1.41** | **96.36%** | **0.00%** |
| rs750222754 | **I388T** | **0.05** | **0.99995** | **99.78** | **0.72** | **-0.27** | **0.49** | **1.43** | **0.31** | **0.83** | **0.83** | **5veu** | **1.12** | **1.41** | **96.36%** | **0.00%** |
| rs756271054 | **R375G** | **0.05** | **0.99995** | **99.78** | **0.84** | **-0.06** | **0.58** | **1.52** | **0.36** | **0.84** | **0.83** | **5veu** | **1.05** | **1.04** | **96.36%** | **0.21%** |
| rs779306884 | **L364H** | **0.05** | **0.99995** | **99.78** | **0.82** | **-0.15** | **0.47** | **1.47** | **0.37** | **0.84** | **0.83** | **5veu** | **1.02** | **1.03** | **96.57%** | **0.00%** |
| rs1245832664 | **E362G** | **0.05** | **0.99995** | **99.78** | **0.75** | **-0.15** | **0.54** | **1.59** | **0.27** | **0.83** | **0.83** | **5veu** | **1.12** | **1.54** | **96.57%** | **0.00%** |
| rs149888520 | **Y355C** | **0.05** | **0.99995** | **99.78** | **0.82** | **-0.10** | **0.43** | **1.56** | **0.34** | **0.83** | **0.83** | **5veu** | **1.12** | **1.41** | **96.36%** | **0.00%** |
| rs1219950418 | **Q352P** | **0.07** | **0.99991** | **99.78** | **0.48** | **0.15** | **0.5** | **1.46** | **-0.03** | **0.83** | **0.83** | **5veu** | **1.15** | **1.41** | **95.93%** | **0.00%** |
| rs1363320186 | **Y347C** | **0.05** | **0.99995** | **99.78** | **0.80** | **-0.03** | **0.54** | **1.42** | **0.34** | **0.84** | **0.83** | **5veu** | **1.12** | **1.41** | **96.36%** | **0.00%** |
| rs990544214 | **I335T** | **0.05** | **0.99995** | **99.78** | **0.76** | **-0.29** | **0.43** | **1.47** | **0.34** | **0.83** | **0.83** | **5veu** | **1.12** | **1.41** | **96.36%** | **0.00%** |
| rs775816439 | **L331P** | **0.07** | **0.99992** | **99.78** | **0.60** | **-0.44** | **0.28** | **1.31** | **0.26** | **0.83** | **0.83** | **5veu** | **1.14** | **1.66** | **96.57%** | **0.00%** |
| rs1489670280 | **K330E** | **0.05** | **0.99995** | **99.78** | **0.84** | **-0.12** | **0.46** | **1.59** | **0.35** | **0.83** | **0.83** | **5veu** | **1.12** | **1.41** | **96.36%** | **0.00%** |
| rs991122268 | **S311R** | **0.06** | **0.99995** | **99.78** | **0.82** | **-0.13** | **0.57** | **1.38** | **0.40** | **0.84** | **0.83** | **5veu** | **1.16** | **1.66** | **96.36%** | **0.00%** |
| rs766695006 | **T309I** | **0.05** | **0.99995** | **99.78** | **0.90** | **0.04** | **0.54** | **1.65** | **0.36** | **0.84** | **0.83** | **5veu** | **0.88** | **0.39** | **96.36%** | **0.00%** |
| rs766695006 | **T309N** | **0.05** | **0.99995** | **99.78** | **0.82** | **0.01** | **0.46** | **1.51** | **0.34** | **0.83** | **0.83** | **5veu** | **0.88** | **0.39** | **96.36%** | **0.00%** |
| rs1562991842 | **E294K** | **0.05** | **0.99995** | **99.78** | **0.75** | **-0.11** | **0.54** | **1.52** | **0.27** | **0.84** | **0.83** | **5veu** | **1.14** | **1.41** | **96.15%** | **0.00%** |
| rs753396001 | **M275R** | **0.11** | **0.99979** | **99.78** | **0** | **-0.23** | **0.73** | **1.43** | **-0.46** | **0.83** | **0.83** | **5veu** | **1.14** | **1.41** | **96.15%** | **0.00%** |
| rs756839053 | **L274P** | **0.06** | **0.99994** | **99.78** | **0.74** | **-0.20** | **0.51** | **1.52** | **0.28** | **0.83** | **0.83** | **5veu** | **1.16** | **1.54** | **96.15%** | **0.00%** |
| rs968643967 | **F248C** | **0.05** | **0.99995** | **99.78** | **0.78** | **-0.23** | **0.53** | **1.46** | **0.35** | **0.84** | **0.83** | **5veu** | **1.12** | **1.41** | **96.36%** | **0.00%** |
| rs979438885 | **S195F** | **0.07** | **0.99992** | **99.78** | **0.79** | **-0.04** | **0.53** | **1.52** | **0.31** | **0.83** | **0.83** | **5veu** | **1.23** | **2.30** | **96.57%** | **0.00%** |
| rs1215839981 | **S195P** | **0.06** | **0.99995** | **99.78** | **0.84** | **-0.13** | **0.47** | **1.53** | **0.37** | **0.83** | **0.83** | **5veu** | **1.19** | **1.54** | **96.36%** | **0.00%** |
| rs781765557 | **F189L** | **0.06** | **0.99995** | **99.78** | **0.75** | **-0.23** | **0.49** | **1.47** | **0.32** | **0.83** | **0.83** | **5veu** | **1.14** | **1.66** | **96.57%** | **0.00%** |
| rs777252753 | **V183G** | **0.05** | **0.99995** | **99.78** | **0.74** | **-0.17** | **0.56** | **1.52** | **0.28** | **0.83** | **0.83** | **5veu** | **1.12** | **1.41** | **96.36%** | **0.00%** |
| rs1584446515 | **D182N** | **0.06** | **0.99995** | **99.78** | **0.85** | **-0.04** | **0.59** | **1.60** | **0.34** | **0.83** | **0.83** | **5veu** | **1.12** | **1.41** | **96.36%** | **0.00%** |
| rs764576184 | **M145T** | **0.05** | **0.99995** | **99.78** | **0.84** | **0.01** | **0.54** | **1.54** | **0.34** | **0.83** | **0.83** | **5veu** | **1.12** | **1.41** | **96.36%** | **0.00%** |
| rs764608096 | **G140R** | **0.05** | **0.99995** | **99.78** | **0.97** | **0.12** | **0.52** | **1.66** | **0.42** | **0.83** | **0.83** | **5veu** | **1.10** | **1.40** | **96.57%** | **0.00%** |
| rs1383200930 | **L133P** | **0.07** | **0.99993** | **99.78** | **0.79** | **-0.09** | **0.37** | **1.40** | **0.36** | **0.83** | **0.83** | **5veu** | **1.14** | **1.54** | **96.36%** | **0.00%** |
| rs758037875 | **R130Q** | **0.05** | **0.99995** | **99.78** | **0.81** | **-0.07** | **0.51** | **1.47** | **0.35** | **0.84** | **0.83** | **5veu** | **1.05** | **1.16** | **96.57%** | **0.21%** |
| rs1294331881 | **W126R** | **0.06** | **0.99995** | **99.78** | **0.81** | **-0.11** | **0.57** | **1.49** | **0.35** | **0.83** | **0.83** | **5veu** | **1.05** | **1.16** | **96.57%** | **0.00%** |
| rs1186517408 | **N104I** | **0.06** | **0.99995** | **99.78** | **0.90** | **-0.25** | **0.41** | **1.48** | **0.47** | **0.83** | **0.83** | **5veu** | **1.12** | **1.66** | **96.79%** | **0.00%** |
| rs761882111 | **T103K** | **0.06** | **0.99995** | **99.78** | **0.78** | **-0.25** | **0.46** | **1.43** | **0.37** | **0.83** | **0.83** | **5veu** | **1.12** | **1.53** | **96.57%** | **0.00%** |
| rs752003788 | **L94Q** | **0.05** | **0.99995** | **99.78** | **0.80** | **-0.11** | **0.53** | **1.43** | **0.36** | **0.84** | **0.83** | **5veu** | **1.12** | **1.41** | **96.36%** | **0.00%** |
| rs56244447 | **L82R (CYP3A5*3D)** | **0.06** | **0.99995** | **99.78** | **0.77** | **-0.19** | **0.46** | **1.49** | **0.33** | **0.83** | **0.83** | **5veu** | **1.16** | **1.79** | **96.57%** | **0.00%** |

| Table S15. All docking Poses RMSD values for the 3 drugs with all 47 CYP3A4 missense SNPs. SNPs highlighted in red recorded the lowest docking S score of the ligand. | | | | |
| --- | --- | --- | --- | --- |
| Variant ID | MUTATIONS | Remdesivir | Nirmatrelvir | Ritonavir |
| rs1256858657 | C468W | 7.848 | 7.219 | 8.529 |
| rs1425544636 | C468Y | 9.427 | 6.331 | 7.837 |
| rs71583803 | F463C | 9.514 | 7.054 | 8.308 |
| rs774168721 | K453N | 9.784 | 5.700 | 6.474 |
| rs1162067586 | G444A | 5.415 | 6.682 | 7.455 |
| rs567089575 | N441D | 7.739 | 9.225 | 8.890 |
| rs1457998170 | P439S | 6.008 | 5.077 | 8.678 |
| rs1166211708 | G438V | 10.219 | 7.892 | 9.8467 |
| rs755248651 | G436R | 6.039 | 5.216 | 6.916 |
| rs1355522141 | P434A | 5.436 | 5.144 | 8.844 |
| rs1368745625 | **R418T** | 8.8028 | 9.3409 | **7.8133** |
| rs4986909 | P416L (CYP3A4*13) | 7.677 | 6.240 | 9.255 |
| rs4986909 | P416R | 7.787 | 5.695 | 8.227 |
| rs72552797 | P411L | 9.986 | 8.104 | 9.598 |
| rs1217252102 | P411A | 7.313 | 5.001 | 7.469 |
| rs1044764678 | W408R | 5.358 | 5.916 | 7.537 |
| rs113716682 | L401P | 7.562 | 6.107 | 8.659 |
| rs1481942841 | P397L | 9.038 | 5.37 | 7.435 |
| rs1195408117 | L364F | 9.83 | 5.071 | 8.086 |
| rs71581998 | V359E | 4.489 | 5.383 | 8.006 |
| rs754968125 | M358V | 7.738 | 4.070 | 8.777 |
| rs1201319750 | Y355C | 8.407 | 6.713 | 8.200 |
| rs1462817145 | Y355H | 9.930 | 7.060 | 9.286 |
| rs368296206 | **I335T (CYP3A4*32)** | 7.728 | **6.484** | 8.576 |
| rs867315029 | E334K | 6.394 | 5.27 | 7.888 |
| rs201821708 | Y319C (CYP3A4*21) | 9.751 | 6.000 | 8.401 |
| rs190354371 | L314P | 6.981 | 3.761 | 8.197 |
| rs267601666 | L314F | 6.590 | 5.400 | 9.497 |
| rs751246524 | T310M | 10.063 | 7.264 | 8.823 |
| rs375997724 | T309I | 4.196 | 10.312 | 8.595 |
| rs71581996 | A305S | 9.625 | 5.931 | 8.045 |
| rs1368114928 | L272P | 8.159 | 6.082 | 8.768 |
| rs1166537703 | L229R | 7.450 | 5.029 | 8.233 |
| rs1467852216 | P227L | 6.309 | 5.530 | 8.977 |
| rs4987161 | F189S (CYP3A4*17) | 7.806 | 5.923 | 8.192 |
| rs773989431 | Y179S | 5.685 | 8.410 | 9.370 |
| rs1396501606 | K173E | 9.977 | 8.523 | 8.620 |
| rs1483230173 | P135L | 5.947 | 3.508 | 9.229 |
| rs72552799 | **R130P** | **9.538** | 8.932 | 8.777 |
| rs72552799 | R130Q (CYP3A4*8) | 9.388 | 5.917 | 8.565 |
| rs778013004 | R130G | 8.662 | 5.743 | 8.399 |
| rs1043569086 | K127N | 9.532 | 7.607 | 8.969 |
| rs142296281 | R105W | 9.757 | 7.358 | 8.387 |
| rs3091339 | K96E | 7.740 | 6.052 | 8.657 |
| rs1194211831 | I90T | 8.452 | 7.559 | 8.321 |
| rs59418896 | Y68C | 7.695 | 6.025 | 8.896 |
| rs760951972 | P39L | 10.580 | 6.401 | 8.302 |

| Table S16. All docking Poses RMSD values for the 3 drugs with all 47 CYP3A5 missense SNPs. SNPs highlighted in red recorded the lowest docking S score of the ligand. | | | | |
| --- | --- | --- | --- | --- |
| Variant ID | MUTATIONS | Remdesivir | Nirmatrelvir | Ritonavir |
| rs777196351 | C467R | 9.729 | 3.916 | 12.105 |
| rs147472467 | A447V | 9.908 | 6.381 | 3.955 |
| rs41279854 | F446S (CYP3A5*10) | 7.196 | 7.339 | 6.891 |
| rs377454308 | G443S | 8.537 | 6.390 | 11.453 |
| rs1267703650 | I442T | 6.157 | 6.466 | 5.630 |
| rs13220949 | R439K | 9.715 | 7.429 | 5.584 |
| rs1035394246 | G435E | 7.851 | 11.582 | 8.075 |
| rs780390510 | G435R | 9.072 | 4.826 | 4.518 |
| rs746993664 | Y431N | 9.739 | 6.939 | 12.003 |
| rs1474237861 | F419L | 1.698 | 6.334 | 3.606 |
| rs370299887 | R418M | 7.491 | 9.665 | 10.811 |
| rs140521496 | P416S | 11.098 | 6.323 | 7.127 |
| rs1299406057 | P411R | 9.722 | 6.182 | 6.257 |
| rs756677833 | P411S | 8.060 | 7.839 | 5.134 |
| rs1562985172 | D404Y | 9.834 | 7.442 | 5.837 |
| rs1252465240 | L401P | 6.520 | 7.448 | 5.481 |
| rs750222754 | I388T | 9.663 | 5.762 | 11.601 |
| rs756271054 | R375G | 6.637 | 5.614 | 6.804 |
| rs779306884 | L364H | 10.724 | 5.331 | 10.451 |
| rs1245832664 | E362G | 8.415 | 4.335 | 4.743 |
| rs149888520 | Y355C | 7.851 | 7.886 | 7.540 |
| rs1219950418 | Q352P | 10.871 | 6.726 | 6.572 |
| rs1363320186 | Y347C | 7.999 | 7.976 | 4.171 |
| rs990544214 | **I335T** | 8.699 | **3.918** | 5.340 |
| rs775816439 | L331P | 11.136 | 9.354 | 11.769 |
| rs1489670280 | K330E | 10.576 | 7.339 | 4.463 |
| rs991122268 | S311R | 8.351 | 6.319 | 11.247 |
| rs766695006 | T309I | 10.007 | 5.762 | 7.320 |
| rs766695006 | T309N | 8.024 | 7.024 | 6.246 |
| rs1562991842 | E294K | 10.886 | 6.202 | 4.656 |
| rs753396001 | M275R | 3.263 | 7.505 | 4.575 |
| rs756839053 | L274P | 10.862 | 5.555 | 11.469 |
| rs968643967 | F248C | 9.303 | 6.615 | 6.162 |
| rs979438885 | S195F | 7.191 | 6.271 | 5.779 |
| rs1215839981 | S195P | 5.832 | 6.461 | 7.201 |
| rs781765557 | F189L | 9.172 | 5.673 | 5.570 |
| rs777252753 | V183G | 10.119 | 6.339 | 4.630 |
| rs1584446515 | D182N | 8.628 | 4.309 | 5.485 |
| rs764576184 | M145T | 10.735 | 9.914 | 2.769 |
| rs764608096 | G140R | 9.511 | 4.850 | 5.640 |
| rs1383200930 | **L133P** | **8.057** | 10.035 | 4.348 |
| rs758037875 | **R130Q** | 6.016 | 7.428 | **6.407** |
| rs1294331881 | W126R | 10.772 | 10.455 | 5.324 |
| rs1186517408 | N104I | 9.232 | 5.763 | 4.424 |
| rs761882111 | T103K | 7.749 | 9.810 | 5.428 |
| rs752003788 | L94Q | 9.792 | 10.645 | 11.208 |
| rs56244447 | L82R(CYP3A5*3D) | 8.942 | 6.718 | 7.625 |

| Table S17. PCR Primer pairs for the most deleterious missense SNPs in CYP3A4/5 identified in this study. | | | | | | | | | |
| --- | --- | --- | --- | --- | --- | --- | --- | --- | --- |
| 1.CYP3A4- R418T- rs1368745625 (99762041 G to C) Primer pair | | | | | | | | | |
|  | **Sequence (5'->3')** | **Template strand** | **Length** | **Start** | **Stop** | **Tm** | **GC%** | **Self-complementarity** | **Self 3' complementarity** |
| Forward primer | CCAGGGGCCTTGTACCTTTC | **Plus** | **20** | **99762026** | **99762045** | **60.32** | **60.00** | **5.00** | **0.00** |
| Reverse primer | CTCTTCACCGTGACCCAAAGT | **Minus** | **21** | **99762095** | **99762075** | **60.20** | **52.38** | **7.00** | **1.00** |
| Product length | **70** | | | | | | | | |
| 2.CYP3A4 - I335T - rs368296206 (99763877 A to G) Primer pair | | | | | | | | | |
|  | **Sequence (5'->3')** | **Template strand** | **Length** | **Start** | **Stop** | **Tm** | **GC%** | **Self-complementarity** | **Self 3' complementarity** |
| Forward primer | TCCATGTACCATCCACTCACC | **Plus** | **21** | **99763835** | **99763855** | **59.16** | **52.38** | **4.00** | **0.00** |
| Reverse primer | TATGAACTGGCCACTCACCC | **Minus** | **20** | **99763926** | **99763907** | **59.38** | **55.00** | **6.00** | **0.00** |
| Product length | **92** | | | | | | | | |
| 3.CYP3A4 - R130P - rs72552799 (99770165 C to T) Primer pair | | | | | | | | | |
|  | **Sequence (5'->3')** | **Template strand** | **Length** | **Start** | **Stop** | **Tm** | **GC%** | **Self-complementarity** | **Self 3' complementarity** |
| Forward primer | TTCATACCTCCTTGAGTTTTCCACT | **Plus** | **25** | **99770115** | **99770139** | **59.93** | **40.00** | **3.00** | **1.00** |
| Reverse primer | GGATGAAGAATGGAAGAGATTACG | **Minus** | **24** | **99770188** | **99770165** | **57.22** | **41.67** | **3.00** | **2.00** |
| Product length | **74** | | | | | | | | |
| 4.CYP3A5- I335T - rs990544214 (99660521 A to G) Primer pair | | | | | | | | | |
|  | **Sequence (5'->3')** | **Template strand** | **Length** | **Start** | **Stop** | **Tm** | **GC%** | **Self-complementarity** | **Self 3' complementarity** |
| Forward primer | CCTTCATCTCCAGGGGTCATC | **Plus** | **21** | **99660471** | **99660491** | **59.58** | **57.14** | **3.00** | **1.00** |
| Reverse primer | TATGAACTGGCCACTCACCC | **Minus** | **20** | **99660570** | **99660551** | **59.38** | **55.00** | **6.00** | **0.00** |
| Product length | **100** | | | | | | | | |
| 5.CYP3A5- L133P - rs1383200930 (99666986 G to A) Primer pair | | | | | | | | | |
|  | **Sequence (5'->3')** | **Template strand** | **Length** | **Start** | **Stop** | **Tm** | **GC%** | **Self-complementarity** | **Self 3' complementarity** |
| Forward primer | TTTTCATACCTCCTTGAGTTTTCC | **Plus** | **24** | **99666943** | **99666966** | **57.11** | **37.50** | **3.00** | **0.00** |
| Reverse primer | AGAATGGAAGAGAATACGGTCAT | **Minus** | **23** | **99667012** | **99666990** | **57.12** | **39.13** | **3.00** | **3.00** |
| Product length | **70** | | | | | | | | |
| 6.CYP3A5- R130Q - rs758037875 (99666995 C to T) Primer pair | | | | | | | | | |
|  | **Sequence (5'->3')** | **Template strand** | **Length** | **Start** | **Stop** | **Tm** | **GC%** | **Self-complementarity** | **Self 3' complementarity** |
| Forward primer | TCCTTGAGTTTTCCGCTGGT | **Plus** | **20** | **99666953** | **99666972** | **59.53** | **50.00** | **2.00** | **0.00** |
| Reverse primer | AGCTGAGGATGAAGAATGGAAGA | **Minus** | **23** | **99667024** | **99667002** | **59.22** | **43.48** | **4.00** | **0.00** |
| Product length | **72** | | | | | | | | |
